# Organellar phylogenomics of Ophioglossaceae fern genera

**DOI:** 10.3389/fpls.2023.1294716

**Published:** 2024-01-15

**Authors:** Li-Yaung Kuo, Huei-Jiun Su, Darina Koubínová, Pei-Jun Xie, Christopher Whitehouse, Atsushi Ebihara, Jason R. Grant

**Affiliations:** ^1^ Institute of Molecular & Cellular Biology, National Tsing Hua University, Hsinchu, Taiwan; ^2^ Department of Earth and Life Sciences, University of Taipei, Taipei, Taiwan; ^3^ University of Neuchâtel, Laboratory of Evolutionary Genetics, Neuchâtel, Switzerland; ^4^ Phillipskop Mountain Reserve, Western Cape, Stanford, South Africa; ^5^ Department of Botany, National Museum of Nature and Science, Tsukuba, Japan

**Keywords:** horizontal gene transfer, mitogenome, MORFFO, Ophioglossaceae, *Rhizoglossum*, phylogenomic, plastome, Santalales

## Abstract

Previous phylogenies showed conflicting relationships among the subfamilies and genera within the fern family Ophioglossaceae. However, their classification remains unsettled where contrasting classifications recognize four to 15 genera. Since these treatments are mostly based on phylogenetic evidence using limited, plastid-only loci, a phylogenomic understanding is actually necessary to provide conclusive insight into the systematics of the genera. In this study, we have therefore compiled datasets with the broadest sampling of Ophioglossaceae genera to date, including all fifteen currently recognized genera, especially for the first time the South African endemic genus *Rhizoglossum*. Notably, our comprehensive phylogenomic matrix is based on both plastome and mitogenome genes. Inferred from the coding sequences of 83 plastid and 37 mitochondrial genes, a strongly supported topology for these subfamilies is presented, and is established by analyses using different partitioning approaches and substitution models. At the generic level, most relationships are well resolved except for few within the subfamily Ophioglossoideae. With this new phylogenomic scheme, key morphological and genomic changes were further identified along this backbone. In addition, we confirmed numerous horizontally transferred (HGT) genes in the genera *Botrypus*, *Helminthostachys, Mankyua*, *Sahashia*, and *Sceptridium*. These HGT genes are most likely located in mitogenomes and are predominately donated from angiosperm Santalales or non-Ophioglossaceae ferns. By our in-depth searches of the organellar genomes, we also provided phylogenetic overviews for the plastid and mitochondrial MORFFO genes found in these Ophioglossaceae ferns.

## 1 Introduction

**Figure 1:**
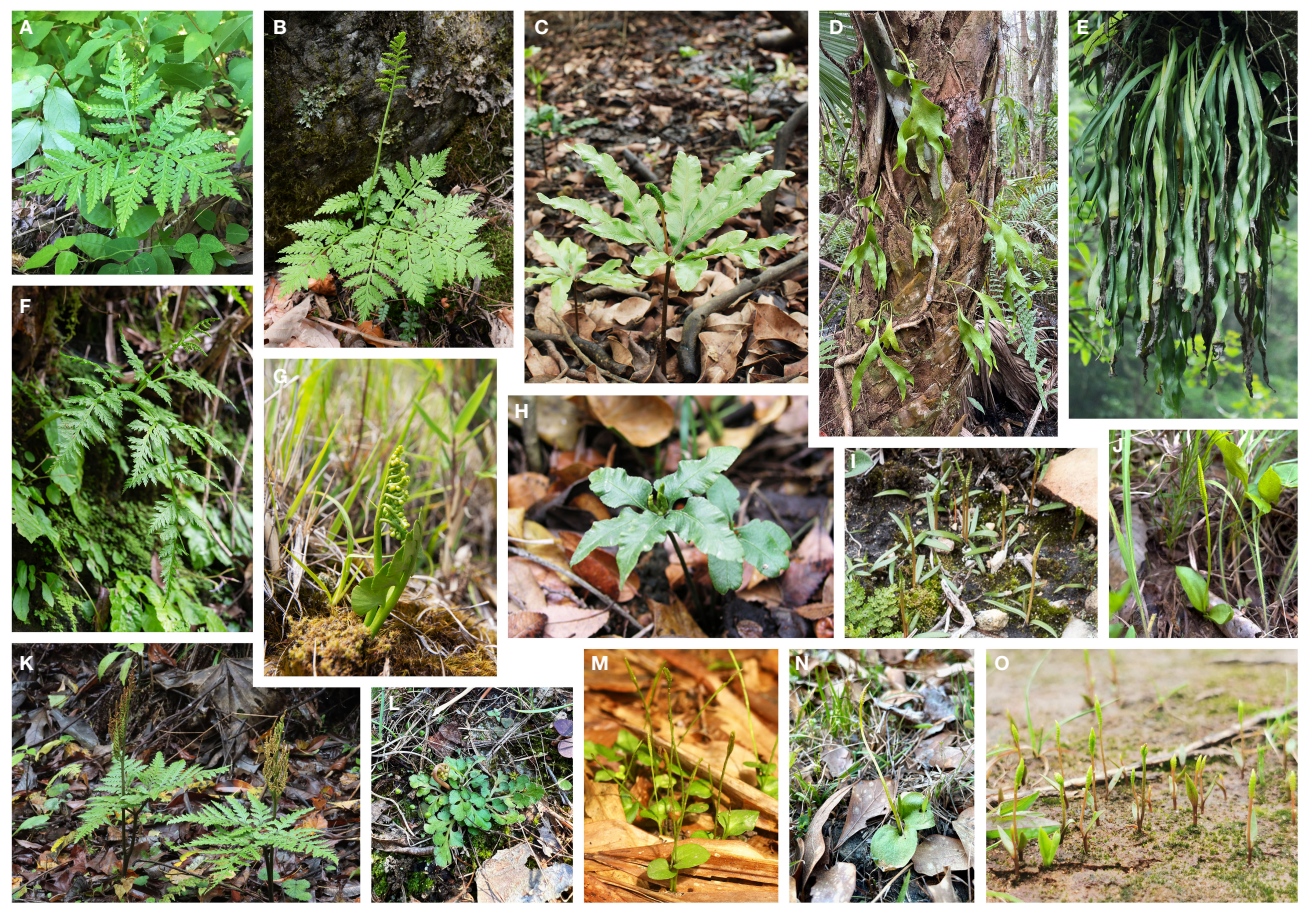
Photos of living plants of Ophioglossaceae genera. **(A)** *Sahashia stricta*. **(B)** *Botrypus virginianus*. **(C)** *Helminthostachys zeylanica*. **(D)** *Cheiroglossa palmata*. **(E)** *Ophioderma pendula*. **(F)** *Japanobotrychium lanuginosum*. **(G)** *Botrychium lunaria*. **(H)** *Mankyua chejuensis*. **(I)** *Rhizoglossum bergianum*. **(J)** *Whittieria engelmannii*. **(K)** *Sceptridium formosanum*. **(L)** *Holubiella lunarioides*. **(M)** *Ophioglossum reticulatum*. **(N)** *Haukia crotalophoroides*. **(O)** *Goswamia isanensis*. Photo credits by Li-Yaung Kuo **(A, K)**, Zhi-Xiang Chang **(B, F, G)**, Tian-Chuan Hsu **(C)**, Emily B. Sessa **(D, L, N)**, Pi-Fong Lu **(E)**, Christopher Whitehouse **(I)**, Ponpipat Limpanasitticha **(M)**, and Tassanai Jaruwattanaphan **(O)**. The photo of *Mankyua chejuensis* **(H)** is modified from Lee et al. (2022).

**Figure 2:**
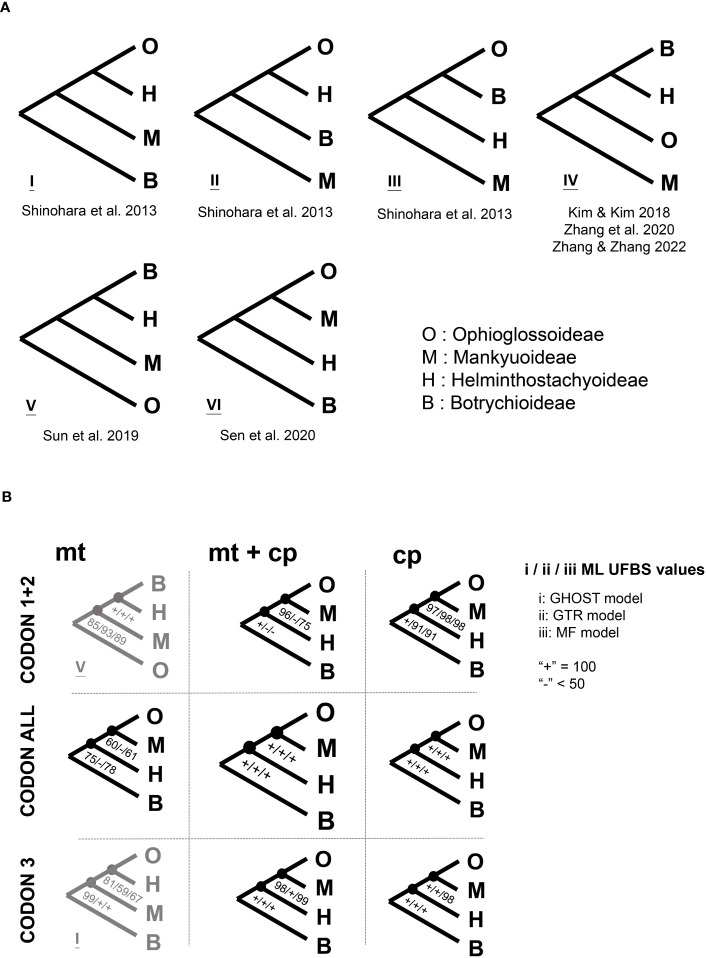
The inter-subfamily relationships of Ophioglossaceae. **(A)** Previous phylogenies, the citations for these phylogenies can be found in “References” of the main text. **(B)** The phylogenies inferred in this study using different models and datasets. Details of the different applied models and datasets can be found in “Materials and Methods” of the main text. ML UFBS, maximum likelihood ultrafast bootstrap.

In ferns, the family Ophioglossaceae (adder’s tongues) is the richest in species among the extant eusporangiate lineages, with over 40% of the species (PPG I, 2016; ~65% estimated by Zhang and Zhang, 2022). Members in this family are highly diversified not only in extrinsic morphology (**Figure 1**) with body size ranging from 1 cm up to 2 m, leaves simple to 4-pinnatifid (Wagner, 1990; Patel and Reddy, 2018), but also intrinsically in their genomic contents with haploid chromosome numbers ranging from 44 to more than 500 (Khandelwal, 1990; **Table 1**), and genome sizes from 6 to > 90 Gbp/C (Kuo et al., 2021). Despite awareness of this disparity within the fern tree of life, the infra-family phylogeny of Ophioglossaceae remains largely unsettled. Due to extreme scarcity of fossils found in the Ophioglossaceae (Rothwell and Stockey, 1989), our evolutionary understanding of this ancient family is built on the phylogeny of solely extant taxa that are currently classified into four subfamilies (PPG I, 2016; Zhang et al., 2020; Zhang and Zhang, 2022). Among these subfamilies, Ophioglossoideae and Botrychioideae are species rich, whereas the remaining two, Helminthostachyoideae and Mankyuoideae are monotypic, each composed by a single species, *Helminthostachys zeylanica* (L.) Hook. and *Mankyua chejuense* B.Y.Sun, M.H.Kim & C.H.Ki. These two orphan lineages are deeply rooted in the Ophioglossaceae phylogeny but change positions in different analyses (**Figure 2A**). *Helminthostachys* had been revealed as the sister of Botrychioideae in many previous phylogenies (Hauk et al., 2003; Sun et al., 2009; Shinohara et al., 2013; Kim and Kim, 2018; Zhang et al., 2020), but in others, as the sister to *Mankyua* + Ophioglossoideae or Botrychioideae + Ophioglossoideae (Shinohara et al., 2013; Shen et al., 2020). Similarly, in these trees, *Mankyua* was placed in different positions that were sister to either Ophioglossoideae, *Helminthostachys* + Ophioglossoideae, *Helminthostachys* + Botrychioideae, or all the remaining subfamilies (**Figure 2A**).

**Table 1:** Morphological and genomic features of Ophioglossaceae genera.

Subfamily	Genera (species number)^a^	Tophophore blade	Vascular cambium	Venation	Sporoophophore blade	Sporangia	Root	Chromosome basic number^b^	Genome size (Gbp/C)^c^
Botrychioideae	*Sahashia* (1)	Divided		Free	Divided	Free, longtitidinal/subtransver dehiscence	Non-budded	44	6.0
Botrychioideae	*Botrypus* (1)	Divided		Free	Divided	Free, longtitidinal/subtransver dehiscence	Non-budded	46 (92)	9.3-11.3
Botrychioideae	*Botrychium* (35)	Divided	Presence	Free	Divided	Free, horizontal dehiscence	Non-budded	45	10.8-28.6
Botrychioideae	*Japanobotrychium* (1)	Divided	Presence	Free	Divided	Free, horizontal dehiscence	Non-budded	45 (90)	14.1-18.6
Botrychioideae	*Holubiella* (1)	Divided	Presence	Free	Divided	Free, horizontal dehiscence	Non-budded	45	9.1-9.4
Botrychioideae	*Sceptridium* (24)	Divided	Presence	Free	Divided	Free, horizontal dehiscence	Non-budded	45	7.8-30.6
Helminthostachyoideae	*Helminthostachys* (1)	Divided	Absence	Free, seldomly anastomosing	Simple	Free, horizontal dehiscence	Non-budded	94	11.7-13.1
Mankyuoideae	*Mankyua* (1)	Divided		Free	Simple or divided	Sunken, basal fused, horizontal dehiscence	Budded	130	12.7
Ophioglossoideae	*Ophioglossum* (~50)	Simple	Absence	Anastomosing	Simple	Sunken, basal fused, horizontal dehiscence	Budded	120	24.5-128.2
Ophioglossoideae	*Haukia* (2)	Simple	Absence?	Anastomosing	Simple	Sunken, basal fused, horizontal dehiscence	Non-budded		18.54
Ophioglossoideae	*Whittieria* (1)	Simple	Absence?	Anastomosing	Simple	Sunken, basal fused, horizontal dehiscence	Budded		16.29
Ophioglossoideae	*Rhizoglossum* (1)	Simple	Absence?	Anastomosing	Simple	Sunken, basal fused, horizontal dehiscence	Budded		
Ophioglossoideae	*Goswamia* (15)	Simple	Absence?	Anastomosing	Simple	Sunken, basal fused, horizontal dehiscence	Budded	120 (86)?	18.61
Ophioglossoideae	*Cheiroglossa* (2)	Simple	Absence?	Anastomosing	Simple	Sunken, basal fused, horizontal dehiscence	Budded		53.5
Ophioglossoideae	*Ophioderma* (6)	Simple	Absence?	Anastomosing	Simple	Sunken, basal fused, horizontal dehiscence	Budded	370-380	98.8-136.7

a based on PPG 1, Zhang et al. (2020) and Zhang and Zhang (2022).

b reviewed in Shinohara et al., 2013, CCDB (http://ccdb.tau.ac.il/).

c reviewed in Kuo et al (2021) unpublished) and Fujiwara et al., 2023.

Morphological characters are based on Hauk et al. (2003) and Zhang and Zhang (2022).

Notably, previous attempts to infer the molecular phylogeny of Ophioglossaceae are all based on plastid sequences. The majority included limited nucleotide sites from no more than six genetic loci and received weak branch supports regarding to the relationships mentioned above. A few of these studies used plastomic datasets for phylogenetic reconstructions, but still revealed conflicting topologies (Kim and Kim, 2018; Shen et al., 2020; Zhang and Zhang, 2022). They all applied a rather simplified nucleotide substitution model, which appears to violate the heterogeneous evolution of loci or codon positions, and thus potentially misleads phylogenetic inference (Kapli et al., 2020). Moreover, these phylogenomic analyses included few (only one or two) representatives for the most species rich subfamilies. These incongruences at the subfamily and genus levels also imply a branch attraction issue for these deep divergences in Ophioglossaceae, in which a phylogenetic inference is believed to be sensitive to homoplasy, model violation, and insufficient sampling (Yang and Rannala, 2012; Kapli et al., 2020). In addition to tackling these difficult nodes, supporting evidence from other genomic features and morphological characters is warranted, and worthy of further investigation.

One of the intriguing findings from Ophioglossaceae is putative intracellular gene transfer (IGT) and horizontal gene transfer (HGT) (Davis et al., 2005; Kim and Kim, 2018; Hao et al., 2022b), and this brings ferns into the earliest foci to study the evolutionary significance of such a genetic mechanism in plants (Li et al., 2014; Li et al., 2018; Wickell and Li, 2020). In particular, angiosperm-to-fern HGTs have been so far discovered only in Ophioglossaceae (Davis et al., 2005). However, for these hypothesized IGTs and HGTs in Ophioglossaceae, their phylogenetic origins or genomic locations remain uncertain. In the case discovered in *Mankyua*, six open reading frames (ORF) had been identified within the *rps4-trnL* intergenic spacer of the plastome (Kim and Kim, 2018). Some of these ORFs were found in plastomes of other Ophioglossaceae but also mitogenome of Ophioglossaceae and plastome of phylogenetically unrelated ferns (Kim and Kim, 2018). Surprisingly, these ORFs were revealed in different locations in these fern plastomes. In addition, some bacteria genomes were blast-matched with sequences similar to these ORFs. Robison et al. (2018) also found putative HGTs with very similar behaviors from different fern plastomes, and named these genes as Mobile Open Reading Frames in Fern Organelles (MORFFO). Two other HGT cases in Ophioglossaceae are found in *Botrypus* (subfamily Botrychioideae). It was found that two (*matR* and *nad1B-C*) of the four mitochondrial gene regions amplified by PCR were grouped with the Loranthaceae within the angiosperm order Santalales, suggesting the gene sequences might be originated from the root-parasitic Loranthaceae (Davis et al., 2005). The genomic origin and location of these *matR* and *nad1* HGTs in *Botrypus* are not yet confirmed. Lacking comprehensive examination of all genomic parts and close relatives, it remains difficult to identify all these putative IGTs and HGTs in Ophioglossaceae and reconstruct a detailed evolutionary scenario for these genes.

In this study, we first aimed to clarify relationships among the four subfamilies and all 15 genera within Ophioglossaceae (**Figure 1**). To infer these deepest divergences, a phylogenomic dataset was compiled by sampling additional representatives from these subfamilies and incorporating loci from not only plastomes but also mitogenomes. Moreover, we applied considerable substitution models for phylogenomic inference to minimize systematic errors that may result from a model simplification or a model violation in a phylogenomic dataset. Next, we aimed to infer the phylogenetic origins of putative IGTs and HGTs reported in Ophioglossaceae. We searched their sequences among Ophioglossaceae genome assemblies representing different genomic parts (i.e., plastome, mitogenome, or nuclear genome) in order to verify their genomic origins. By blasting broadly against the nucleotide collection of GenBank and our genome assemblies, we then gathered all highly matched sequences into the phylogenetic surveys of these putative IGTs and HGTs. Moreover, the phylogenomic backbone inferred here provides a solid basis for us to track the evolutionary origins of these putative IGTs and HGTs in Ophioglossaceae as well as the morphological features and genomic changes in this family.

## 2 Materials and methods

### 2.1 Sampling and assembling organellar genomes

In total, we included 20 taxa for phylogenomic analyses, covering all four subfamilies and 15 genera in Ophioglossaceae (PPG I, 2016; Zhang and Zhang, 2022) as well as four outgroup taxa. Nine Ophioglossaceae collections were first sequenced in this study, and the others were from previous works (**Table S1**). To generate new genome skimming data, the DNAs were first extracted using a modified CTAB protocol (Kuo, 2015), and then fragmented in a Covaris S2 ultrasonicator (Covaris, Woburn, MA, USA) with a size range of 300~400 bp if found to be on average longer than this size. The fragmented DNAs were input for illumina library construction using a NEBNext Ultra II DNA Library Prep Kit for Illumina (New England Biolabs, Ipswich, MA, USA), and following the industry manual. They were finally sequenced using NovaSeq or HiSeq 150 PE (illumina, San Diego, CA, USA) with about 3~10 Gbp per sample.

Except for *Ophioglossum californicum* Prantl and the outgroups, which were already published with both complete plastomes and mitogenomes (**Table S1**), we assembled both organellar genomes of the other samples. The illumina reads were first trimmed using fastp (Chen et al., 2018) with the default settings, and the trimmed reads were input for organellar genome assembling using NOVOplasty (Dierckxsens et al., 2017; Dierckxsens et al., 2020) with K=39. The plastomes were first assembled with seeds of conspecific *rbcL* sequences downloaded from GenBank. In some samples, other plastid genes were used as seeds for assembly when these genes were missing from the first version of plastome assembly. To fill gaps among the plastome contigs of *Botrypus*, Sanger sequencing was used, and the PCR primers are detailed in **Table S2**. For the mitogenome assembling of each sample, we used the final plastome assembly from the same collection which could prevent chimeric assemblies with plastid reads (Dierckxsens et al., 2020), and conducted multiple NOVOplasty runs each of which used a different mitochondrial gene as a seed. These seed sequences covered all coding genes (CDS) known from the complete mitogenome sequences of close relatives−*Ophioglossum* and *Psilotum* (Guo et al., 2017). For each sample, we pooled mitogenome assemblies from different NOVOplasty runs, and removed identical sequences prior to annotation.

For gene annotation of both organellar genomes, we used Geneious (Kearse et al., 2012) and published organellar genomes of *Ophioglossum* and *Psilotum* as references (**Table S1**). The parameter of similarity was set to 80% and 60% for plastid and mitochondrial genes. Because of the highly repeated nature of fern mitogenomes (e.g., Feng and Wicke, 2022; Zumkeller et al., 2023), an assembling approach using short reads alone was unlikely to assemble a complete fern mitogenome. Therefore, we additionally carried out *de novo* assembling for all Ophioglossaceae samples using SOAPdenovo2 (Luo et al., 2012) and the trimmed reads were used as input with a K-mer setting of 29. tblastn function of blast-2.10.0 + (Camacho et al., 2009) was used to further inspect the presence of mitochondrial CDS among these NOVOplasty and SOAP assemblies.

Each organellar CDS was then aligned under a codon model by MACSE v2.03 (Ranwez et al., 2011) using a setting of “-max_refine_iter 3 -local_realign_init 0.3 -local_realign_dec 0.2”. We manually checked these resulting alignments, made adjustments if necessary, and removed all ambiguously aligned sectors in the ends of these alignments. For each of them, we finally reconstructed a preliminary gene tree to confirm homology/origin of each sequence. The sequences found to phylogenetically behave like potential horizontal transfers (e.g., not grouping with other Ophioglossaceae sequences) were excluded from the alignments for our phylogenomic analyses.

### 2.2 Phylogenetic analyses

A total of 37 mitochondrial and 83 plastid CDS alignments were concatenated, and then compiled into nine datasets for our phylogenomic analyses, including mitochondrial (mt), plastid (cp), and mitochondrial + plastid (mt + cp). Each of them contained three datasets: with (1) the first two codon positions (CODON 1 + 2), (2) the third codon position (CODON 3), and (3) all three codon positions (CODON ALL). For the mitochondrial part, *ccm* genes were not included because they were found lacking in the mitogenome assembly of certain Ophioglossaceae lineages (Guo et al., 2017; this study). The *Holubiella* sample was excluded from our mitochondrial datasets, because we recovered only a few mitochondrial CDS genes from its assembly. The reads of this herbarium collection were relatively short due to its highly fragmented DNAs, and thus resulted in a poor assembly for the mitogenome. The presence or absence of these CDS in the datasets was summarized in **Table S3**. IQ-TREE v2.1.3 was used to perform our phylogenomic analyses (Nguyen et al., 2015; Minh et al., 2020). For every dataset, we conducted three maximum likelihood (ML) analyses with different models: (1) MF: the best partition scheme and substitution rates inferred by ModelFinder (Kalyaanamoorthy et al., 2017) with “rcluster = 100” and Bayesian information criterion, (2) GTR: the finest partitions every with a GTR+F+R10 model, (3) GHOST: the “General Heterogeneous evolution On a Single Topology” model (Crotty et al., 2020) with six unlinked GTR classes (i.e., GTR*H6). In the first two kinds of analyses, the datasets were first partitioned by codon positions by genes. Each of these phylogenetic analyses was conducted with 1000 ultrafast bootstrap (UFBS) replicates (Hoang et al., 2018). In addition, we analyzed gene and site concordance factors (gCF and sCF; Minh et al., 2020) based on the mt + cp CODON ALL dataset.

### 2.3 Inferring origins of intracellular and horizontal gene transfer

For the sequences suspected to be IGTs or HGTs, we first blasted (blastn and tblastn) them against the nucleotide collection in GenBank and our organellar genome assemblies to explore their possible origins. We then selected sequences that were best matched or the most closely related ones shown in a NCBI BLAST distance tree, and aligned them with our mitochondrial matrices. In addition, we found several mitochondrial HGTs to have an angiosperm origin, especially Santalales. Most other mitochondrial HGTs appeared to have non-Ophioglossaceae fern origins. In order to gain a deeper understanding of their phylogenetic origins, we expanded our mitochondrial matrices to include more representatives from angiosperms, in particular for Santalales (**Table S4**). Besides including all published mitogenomes of this angiosperm order, we also included sequences of certain mitochondrial genes (e.g., *matR* and *nad1B-C*) to cover additional families and genera. By these expanded mitochondrial alignments, we reconstructed their individual gene and locus-concatenated ML phylogenies using the MF model (details same as in earlier) using IQ-TREE. In the expanded alignment of *matR*, several sequences appeared to be a pseudogene. Therefore, we realigned these DNA metrics based on a nucleotide mode using MUSCLE (Edgar, 2004) implemented in AliView (Larsson, 2014). As a result, we set only a single partition (i.e., not to partition it with different codon positions) for the phylogenetic reconstruction with this gene. For *nad1B-C*, we used the same approach for its alignment and phylogenetic analysis, because it was composed mostly of the non-coding region−intron 2. In addition, due to the difficulty of aligning sequences across different vascular plant lineages, we included only angiosperm (i.e., close relatives of the donor) sequences for our phylogeny of this HGT.

To further explore whether these HGT-like sequences were located in mitogenome or not, we also evaluated the read coverages of these HGT-like sequences and compared them with those of the mitochondrial CDS genes. In practice, we input the exon sequences of the mitochondrial CDS that were longer than 100 bp, and also these HGT-like sequences for read mapping using BWA v0.7.17 (Li and Durbin, 2009). We calculated the mean depths of all sequences using the SAMtools function “coverage” (Danecek et al., 2021). By our genome skimming approach of < 10 Gbp per sample, sequence depth of a nuclear gene in these Ophioglossaceae taxa was theoretically far less than two due to their considerably large sizes of nuclear genomes (**Table 1**). Conversely, finding a gene with a higher sequence depth indicated its location in an organellar genome. Once we obtained completed plastome assembly, we could further verify whether such a gene was located in plastome or not. If not found in the plastome, we considered it a mitochondrial gene.

## 3 Results

### 3.1 Genome assembly and gene content

We newly assembled ten plastomes in this study, and the information about their GenBank and SRA accessions is provided in **Table S1**. Each of these plastomes was assembled into a complete and circularized contig, except for that of *Botrypus* and *Holubiella*. These two were composed by one and five linear contigs, respectively. Nonetheless, no plastid genes were missing from their assembly. Notably, compared with the previous plastome by Zhang and Zhang (2022) (GenBank accession: OM897597), our plastome of *Whittieria* was fully assembled without gaps nor sectors of ambiguous bases despite that the same read source was input. Except for the inversion of *trnT-*GGU in *Sceptridium* and *Holubiella*, we found no structural difference among these Ophioglossaceae plastomes, and their gene boundaries between inverted repeat (IR) and large or small single copy regions (LSC or SSC) were also identical. Losses of *psbM*, *trnA*-UGC, *clpP* 2^nd^ intron, and *rpl2* intron were identified in genera of subfamily Ophioglossoideae (**Table S5**).

**Figure 3:**
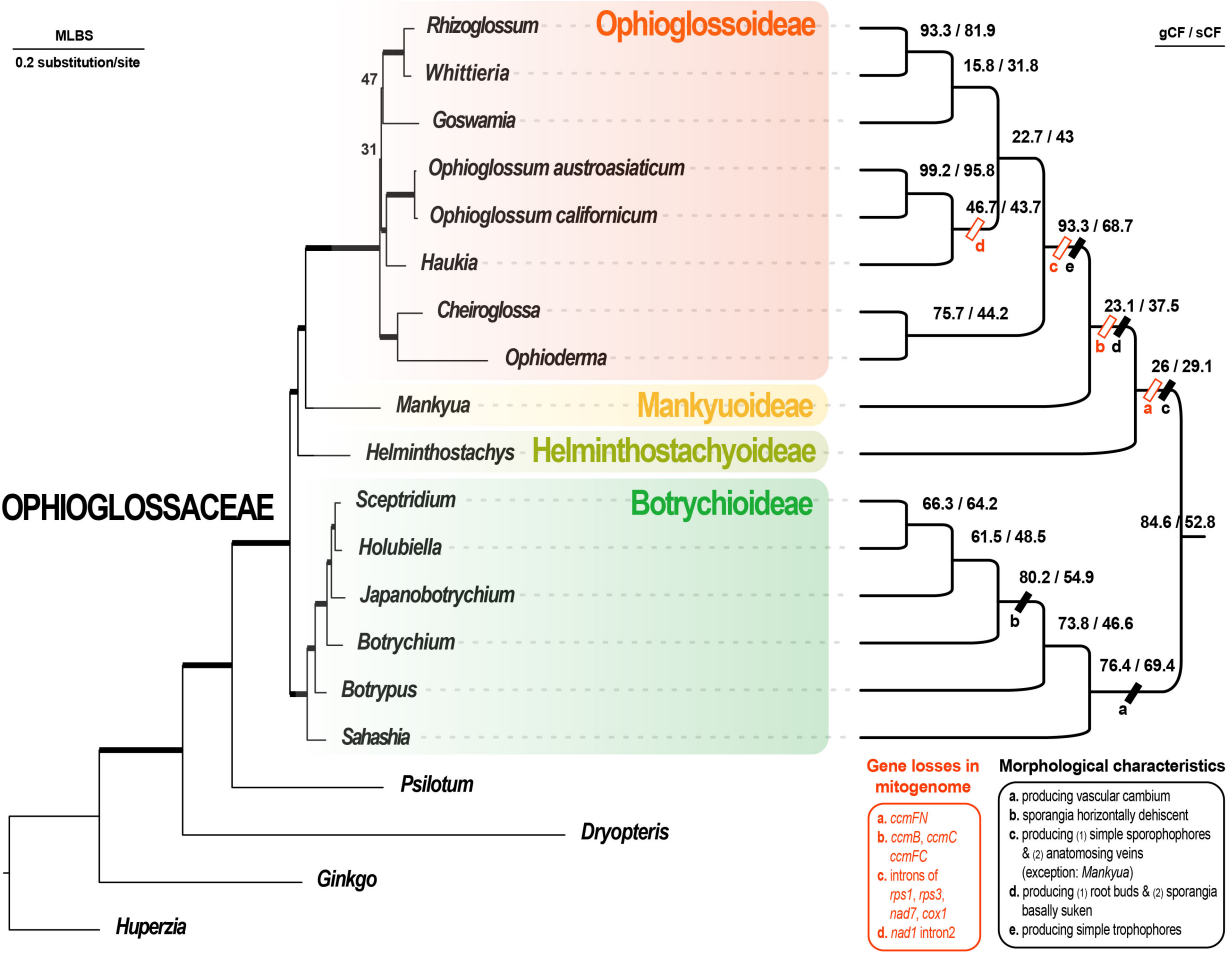
Maximum likelihood phylogenomic tree based on 37 mitochondrial CDS + 83 plastid CDS using the best scheme inferred by ModelFinder. In the phylogram (left part), the branches with ML UFBS (maximum likelihood ultrafast bootstrap) values of 100 are bolded, and only the values less than 100 are shown on the branches. The cladogram (right part) shows the site and gene concordance factors (sCF and gCF) of the branches. The genus-shared morphological and mitogenomic characteristics are also informed on the branches.

Our mitogenome assemblies were rather fragmented and composed of numerous linear contigs from 47 in *Cheiroglossa* to 6,749 in *Ophioderma*. In every sample, mitochondrial genes could be found in multiple contigs that slightly differed in their non-coding sequences (**Supplementary File S1**). Notably, we have further confirmed the loss of introns in several CDS genes (**Table S5**) as well as the loss of *ccm* genes in subfamilies Ophioglossoideae, Mankyuoideae, and Helminthostachyoideae (**Table S5**, **Figure 3**). Slightly differing from Guo et al. (2017), the presence of *ccmC* gene was also confirmed in *Botrypus* and *Sceptridium*. Assembly with annotations is also supplied in **Supplementary File S1**.

### 3.2 Phylogenomic relationships

Our gene alignments (before and after trimming) are provided in **Supplementary File S2**. We summarized our phylogenomic results in **Figures 2**, **3**, **S1**. Our reconstructions revealed a solid backbone at the subfamily-level (**Figure 2B**), and supported well (i.e., ML UFBS values from 90 to 100) the topology of (Botrychioideae, (Helminthostachyoideae, (Mankyuoideae, Ophioglossoideae))). These relationships were only weakly supported in the mt datasets (**Figure 2B**). Notably, this subfamily topology (i.e., topology VI in **Figure 2A**) had not been recovered in previous works, except for Shen et al. (2020) (**Figure 2A**). Within Botrychioideae, one of the two non-monotypic subfamilies, the inter-generic relationships were stable and well resolved with high branch supports (**Figure 3**; data not shown). These relationships are also congruent with previous phylogenies (Hauk et al., 2003; Sun et al., 2009; Shinohara et al., 2013; Zhang et al., 2020; Zhang and Zhang, 2022). In contrast, the inter-generic relationships in another non-monotypic subfamily, Ophioglossoideae, remain poorly resolved (**Figures 3**, **S1**). The seven Ophioglossoideae genera fell into four clades: *Goswamia* (G), *Rhizoglossum* + *Whittieria* (R-W), *Cheiroglossa* + *Ophioderma* (C-O), *Haukia* + *Ophioglossum* (H-O). Although the monophyly of each of these clades is highly supported, the relationships between them remain unclear (**Figures 3**, **S1**). Based on our phylogenomic analyses, we totally identified eight different topologies representing their relationships (**Figure S1**).

### 3.3 Gene sequences with putative IGT or HGT origins

**Figure 4:**
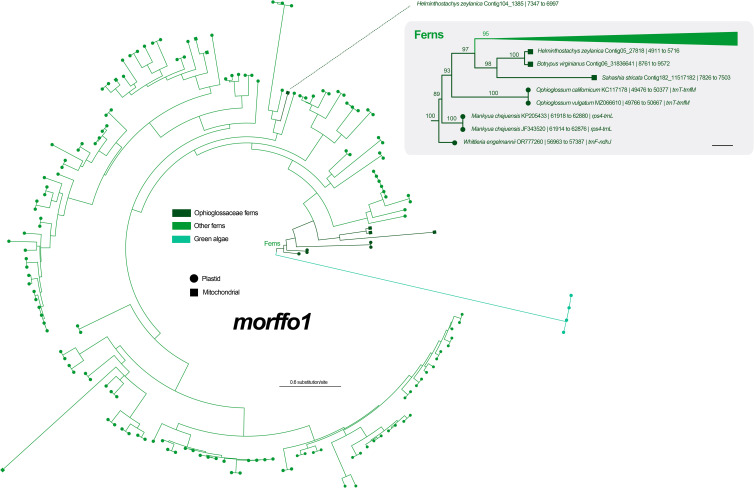
Maximum likelihood (ML) phylogeny of *morffo1* using midpoint rooting. The detailed relationships of Ophioglossaceae sequences are highlighted in the right part, and each tip is presented with information as: species name and GenBank accession number or contig no. | site positions | genic position (only for plastid ones). The values on the branches are their supports from ML ultrafast bootstraps. The bar in the right part = 0.2 substitution per site.

**Figure 5:**
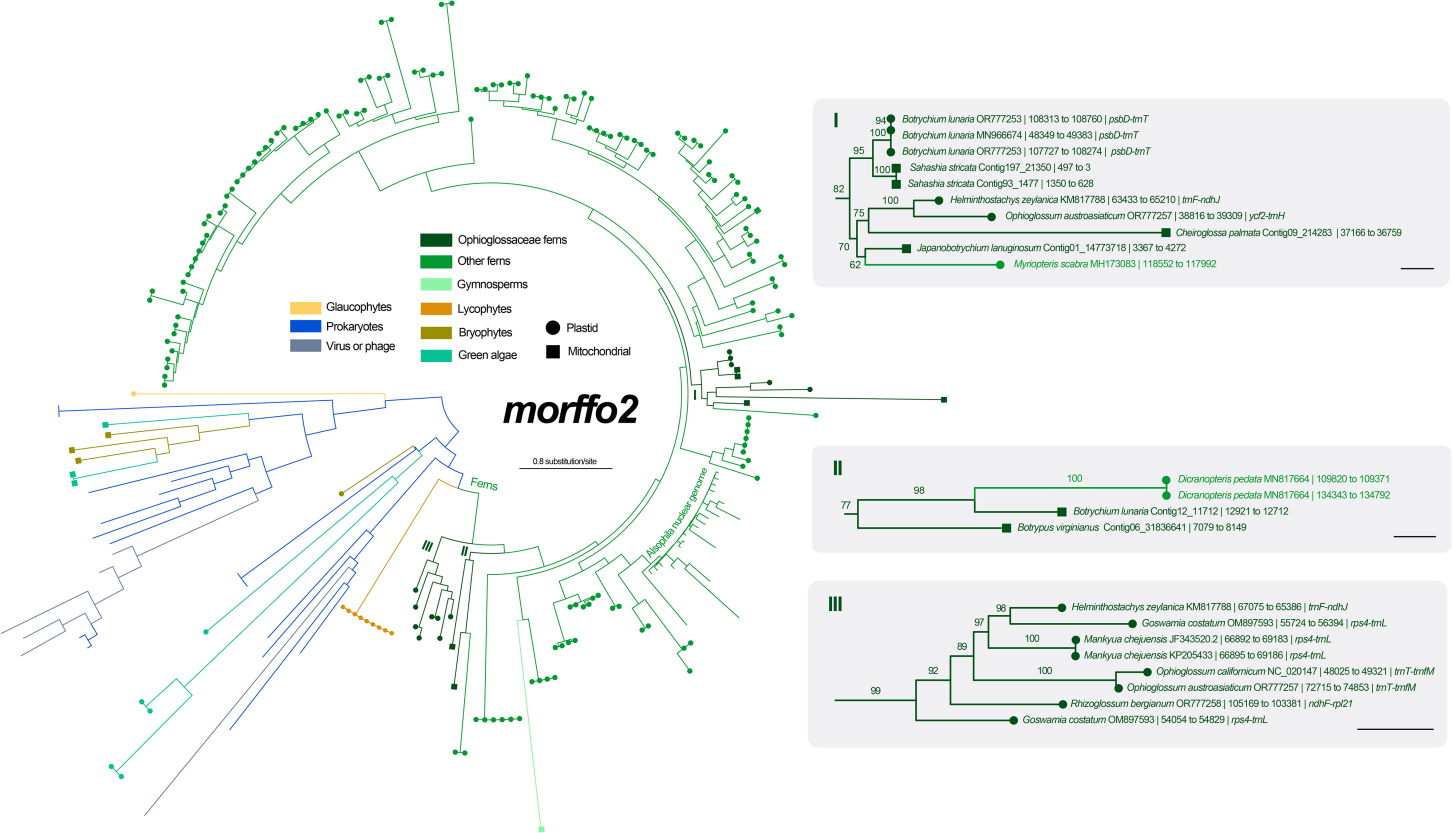
Maximum likelihood (ML) phylogeny of *morffo2* using midpoint rooting. The detailed relationships of Ophioglossaceae sequences are highlighted in the right part, and each tip is presented with information as: species name and GenBank accession number or contig no. | site positions | genic position (only for plastid ones). The values on the branches are their supports from ML ultrafast bootstraps. The bars in the right part = 0.2 substitution per site.

Among six unknown ORFs in the *Mankyua* plastome (Kim and Kim, 2018), we confirmed that three of them are actually homologs of MORFFO genes that were first identified by Robison et al. (2018). ORF 295 belongs to *morffo1*, while ORFs 531 and 187 are parts of *morffo2*. These MORFFO sequences were found in not only the plastomes but also in the mitogenomes of land plants (**Figures S2, S3**). In addition, we had identified these genes in many other Ophioglossaceae genera, including *Sahashia*, *Botrypus*, *Botrychium*, *Japanobotrychium*, *Whittieria*, *Rhizoglossum*, *Cheiroglossa*, *Goswamia*, and *Helminthostachys*. Interestingly, *morffo2* could also be found in non-fern organellar genomes, including those from glaucophytes, green algae, bryophytes, lycophytes, and gymnosperms (**Figure S3**). The phylogenies of *morffo1* and *morffo2* are shown in **Figures 4** , **5** with much more details in **Figures S2, S3**. However, none of Ophioglossaceae species nor any other species had been found with homologs in both its plastome and mitogenome (**Figures 4**, **5**, **S2, S3**). In other words, no evidence indicating an IGT can be found in our MORFFO phylogenies. On the other hand, MORFFO sequences from different fern families and other plant lineages are usually mixed in our phylogenies (**Figures S2, S3**). Such mixture patterns imply frequent HGT among these plant lineages, particularly fern species. ORFs 135, 372, and 436 seem to be specific to the plastome of *Mankyua*. Besides its plastome, we found no confident blast hit for these ORFs through the GenBank’s nucleotide collection and our genome assemblies, except for ORF-436-like sequence found in the *trnT-trnfM* intergenic region in the plastome of *Ophioglossum californicum*, which is also revealed by Kim and Kim (2018). For ORF 135, we found only BLAST matches of short fragments (< 50% of quarry length, and < 150 bp) from other organisms.

**Figure 6:**
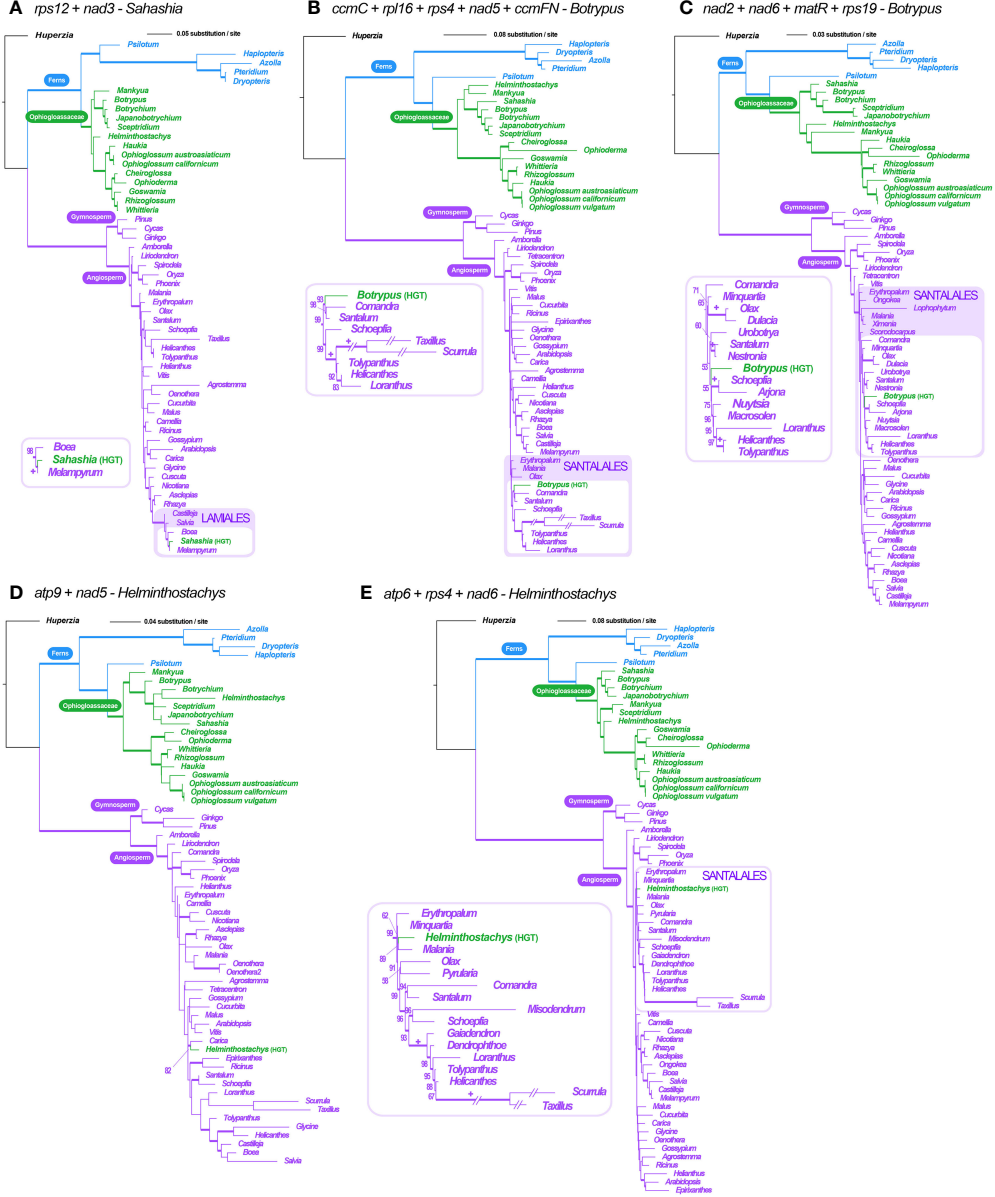
Locus-concatenated phylogenies for the HGT genic clusters found in Ophioglossaceae mitogenomes. **(A)** *rps12* + *nad3* in *Sahashia*. **(B)** *ccmC* + *rpl16* + *rps4* + *nad5* + *ccmFN* **(C)** *nad2* + *nad6* + *matR* + *rps19* in *Botrypus*. **(D)** *atp9* + *nad5* **(E)** *atp6* + *rps4* + *nad6* in *Helminthostachys*. The values below the branches are their ML UFBS, and only values larger than 50 are shown. The branches with supporting values of maximum likelihood ultrafast bootstrap ≥ 80 are bolded. “+” = 100.

In addition to the cases of *matR* and *nad1B-C* reported in *Botrypus* (Davis et al., 2005), we found more mitochondrial HGTs that originated from either angiosperm species or ferns based on our phylogenetic surveys (**Table 2**, **Figures 6**, **S4-23**). We also discovered such mitochondrial HGTs in many other Ophioglossaceae genera, including *Helminthostachys*, *Mankyua*, *Sahashia*, and *Sceptridium*, but none of them was from subfamily Ophioglossoideae (**Table 2**). Moreover, several of these HGTs were identified in clusters on the same contigs, and some were even found to be linked with a host mitochondrial genes (i.e., from the Ophioglossaceae taxon of the sample) (**Table 2**). In the case of *nad1B-C* (i.e., intron 1 with partial exons 2 and 3) HGT in *Botrypus*, we didn’t recover this HGT successfully from our mitogenome nor from plastome NOVOplasty assemblies, but we did find it in a SOAP contig. The read coverages (i.e., sequence depth) of mitochondrial genes ranged from 1.0 to 29.3 per Gbp (**Table S6**). Except for the unusually high coverage of *nad7* exon3 HGT in *Sahashia*, the coverage values of these mitochondrial HGTs also fell into this range (**Table 2**), and support that they are physically located in mitogenomes. However, more than half of these HGTs are pseudogene-like containing frame-shift mutations (**Table 2**).

**Table 2:** Mitochondrial HGT genes found in Ophioglossaceae mitogenomes.

Taxon	Gene	Read coverage per Gbp (total coverage)	HGT origin	Close relative based on ML-tree	Pseudogene-like	Native homolog
*Botrypus*	*ccmC* ^a^	9.00 (26.05)	angiosperm	Santalales (sister to *Tolypanthus/Helicanthus/Taxillus*)	yes	yes
*Botrypus*	*rpl16* ^a^	14.33 (41.45)	angiosperm	Santalales (*Santalum/Comandra*)	yes	not found
*Botrypus*	*rps4* ^a^	9.45 (27.34)	angiosperm	Santalales (sister to *Tolypanthus/Helicanthus*)	yes	yes
*Botrypus*	*nad5* exon1^a^	13.44 (38.89)	angiosperm	unresolved	yes	yes
*Botrypus*	*nad5* exon2^a^	9.76 (28.23)	angiosperm	Santalales	no	yes
*Botrypus*	*ccmFN* ^a^	12.43 (35.96)	angiosperm	Santalales (week support)	yes	yes
*Botrypus*	*nad2* exon4* ^b^	10.04 (29.04)	angiosperm	Santalales (*Comandra*)	yes	yes
*Botrypus*	nad6* ^b^	7.65 (22.13)	angiosperm	Santalales + *Vitis*	yes	yes
*Botrypus*	matR* ^b^	10.51 (30.39)	angiosperm	Santalales (sister to *Schoepfia* + *Arjona*)	yes	yes
*Botrypus*	rps19* ^b^	18.49 (53.50)	angiosperm	unresolved	no	yes
*Botrypus*	*nad1* intron2 (with partial exons 2 & 3)	9.58 (27.72)	angiosperm	Santalales (*Schoepfia*)	yes	yes
*Helminthostachys*	*atp6* ^c^	4.95 (22.94)	angiosperm	Santalales	no	yes
*Helminthostachys*	*rps4* ^c^	4.99 (23.12)	angiosperm	Santalales (*Malania*)	yes	yes
*Helminthostachys*	*nad6* ^c^	4.89 (22.67)	angiosperm	unresolved	no	yes
*Helminthostachys*	*atp9* ^d^	10.88 (50.41)	angiosperm	unresolved	no	yes
*Helminthostachys*	*nad5* exon1^d^	2.33 (10.79)	angiosperm	unresolved	no	yes
*Helminthostachys*	rpl16*	5.12 (23.72)	angiosperm	Santalales (*Olax*)	yes	yes
*Helminthostachys*	*atp9*	11.22 (52.00)	fern	non-Ophioglossaceae ferns	no	yes
*Mankyua*	*rpl5* ^e^	3.62 (59.75)	fern	Ophioglossaceae ferns (*Sceptridium*)	no	yes
*Mankyua*	*ccmB* ^e^	4.11 (67.92)	fern	Ophioglossaceae ferns (*Sceptridium*)	no	not found
*Mankyua*	*nad2* exon4	5.03 (83.11)	angiosperm	unresolved	yes	yes
*Mankyua*	*rps4*	4.29 (70.95)	angiosperm	*Liriodendron*	no	yes
*Sahashia*	ccmC* ^f^	17.92 (62.40)	angiosperm	unresolved	yes	yes
*Sahashia*	rps12* ^f^	10.76 (37.46)	angiosperm	Lamiales (*Melampyrum*)	no	not found
*Sahashia*	nad3* ^f^	10.25 (35.69)	angiosperm	Lamiales (*Melampyrum*)	yes	not found
*Sahashia*	*nad5* exon2 *+* exon3 *+* exon5 *+* exon6	7.92 (27.59) [based on exon2]	angiosperm	Lamiales (*Castilleja*)	no	yes
*Sahashia*	*cox3*	13.34 (46.45)	fern	non-Ophioglossaceae ferns	yes	not found
*Sahashia*	*nad7* exon1 + exon2 + exon3	371.67 (1294.06) [based on exon3]	fern	leptosporangiate ferns	yes	not found
*Sahashia*	*nad7* exon4 + exon5	15.99 (55.68)	fern	leptosporangiate ferns	yes	not found
*Sceptridium*	ccmC*	4.83 (21.78)	angiosperm	unresolved	no	yes

*Linked with a host mitochondrial gene.

a on the contig of “ccmC-rpl16-rps4-nad5-ccmFN”.

b on the contig of “nad2-nad6-nad1e4-matR-rps19”.

c on the contig of “atp6-rps4-nad6”.

d on the contig of “atp9-nad5”.

e on the contig of “rpl5-ccmB”.

f on the contig of “ccmC-rps12-nad3”.

Furthermore, we concatenated the sequences of the HGTs from the same contigs in order to reconstruct their phylogenies and to infer their origins with better resolutions. Both cases in *Botrypus* and one in *Helminthostachys* were shown to be nested in the angiosperm order Santalales (**Figures 6A–C**). Another case in *Helminthostachys* is sister to *Carica* (order Brassicales) (**Figure 6D**), and the case of *Sahashia* is nested in the angiosperm order Lamiales (**Figure 6E**).

## 4 Discussion

### 4.1 Phylogenomic relationships within Ophioglossaceae

Our previous understanding of Ophioglossaceae phylogeny had been based on molecular phylogenetic analyses using limited plastid loci. Although infrafamilial relationships were inferred by plastomic datasets in many recent phylogenies, those studies sampled limited generic representatives and applied rather simplified substitution models in their phylogenomic analyses (Sun et al., 2009; Kim and Kim, 2018; Shen et al., 2020; Zhang and Zhang, 2022). Those previous phylogenies also revealed conflicting relationships (**Figure 2A**). In the current study, we compiled the broadest phylogenomic dataset to date covering all 15 Ophioglossaceae genera and included both their plastomic and mitogenomic sequences. We then set a finer genic partition that allowed a greater complexity of substitution patterns for these phylogenomic datasets that we considered to be more realistic. Using these, we resolved relationships at the subfamily level and revealed a well-supported backbone that was consistent with the majority of our phylogenomic results derived from different datasets and substitution models (**Figures 2**, **3**). Notably, this backbone is also supported by several key morphological and genomic features (**Figure 3**). Mankyuoideae and Ophioglossoideae share the morphological diagnostics of sunken sporangia and budded roots, and both subfamilies lack all *ccm* genes in their mitogenomes (**Figure 3**). Together with Helminthostachyoideae, these three subfamilies possess undivided sporangial spikes and *ccmFn* is lacking in their mitogenomes (**Figure 3**). In addition, they have higher basic chromosome numbers (≥ 94) compared with that in Botrychioideae (**Table 1**). Similarly, their haploid genome sizes (i.e., C-value) are larger (> 11 Gbp/C; **Table 1**). These genomic increases also imply a shared ancient whole genome duplication (WGD) event(s). Since these ferns are polyploidy-prone, their nuclear phylogenies are likely complicated by frequent gene duplication-and-loss events in the nuclear genomes (e.g., inclusion of paralogs; Zhou et al., 2022). Nonetheless, future nuclear phylogenomics, incorporating a comprehensive sampling of genera, holds promise for reconsolidating the aforementioned evolutionary scenarios. Specifically, these can provide further supporting evidences not only for the present topology but also its tree-linked evolutionary episodes, including the trajectory of *ccm* gene losses in nuclear genomes (Guo et al., 2017) and the ancient WGD(s) (Huang et al., 2020).

In previous phylogenies, wobble positions of the two orphan lineages, Helminthostachyoideae and Mankyuoideae, seemingly resulted from a slow substitution manner of analyzed DNA datasets. Because of being early diverged, their internodes in a phylogeny are coupled with relatively short branch lengths, which are sensitive to stochastic and homoplasic signal, and hence difficult to be correctly inferred when a slow-evolving dataset, such as mt CODON 1 + 2 here, is employed for a phylogenetic inference. Likewise, an oversimplified substitution model and insufficient taxon sampling could result in incorrect length inferences. Such systematic errors would subsequently cause branch attraction issues (Yang and Rannala, 2012). The intergeneric relationships within subfamily Ophioglossoideae were still unresolved even though both plastomic and mitogenomic CDS genes were incorporated in current phylogenomic analyses. Overall, these seven genera fell into four well-supported clades (**Figures 2**, **S1**). Some mitogenomic features likely support certain groupings while others remain ambiguous (**Figure 3**; **Table S5**). The grouping for *Ophioderma* and *Cheiroglossa* is highly supported (**Figure 3**), and this solid relationship is also recovered in most previous phylogenies (Hauk et al., 2003; Shinohara et al., 2013; Zhang et al., 2020; Zhang and Zhang, 2022). Morphologically, the two genera share several features, including their unique epiphytic growth form, and the sporangial spikes emerging from the middles of laminae. The grouping of *Haukia* and *Ophioglossum* is also well-supported in our phylogenomic analyses, and this relationship is congruent with Zhang and Zhang (2022). In addition, the two genera also share the mitogenomic losses of *nad1* intron2. Finally, our work is the first one to place *Rhizoglossum* onto a generic phylogeny of Ophioglossaceae (**Figure 3**). It was unambiguously placed as the sister of *Whittieria*, however, we have not found any morphological or genomic synapomorphy to support this sister relationship.

### 4.2 Evolutionary origins of MORFFO genes

MORFFO genes were first identified by Robison et al. (2018) in many fern plastomes, and *morffo1* and *morffo2* were also discovered in Ophioglossaceae plastomes by Kim and Kim (2018) who reported them as ORF 295 and ORFs 531 and 187, respectively. Kim and Kim (2018) also hypothesized their origins in Ophioglossaceae by HGT and/or IGT. In the current study, we blast-searched these MORFFO sequences through both plastome and mitogenome assemblies across different Ophioglossaceae genera. We indeed recovered mitochondrial MORFFO genes from our assemblies (**Figures 4**, **5**, **S2, S3**). Together with blast-matched sequences from the GenBank nucleotide collection, our phylogenies, however, reveal no direct relationships between MORFFO sequences in a plastome and a mitogenome in any certain species (**Figures 4**, **5**, **S2, S3**). Specifically, for either *morffo1* or *morffo2*, we didn’t find its presence in both organellar genomes in the same Ophioglossaceae collection. In other words, our study provided no supporting evidence for IGT of a MORFFO gene that could switch between organellar genomes in Ophioglossaceae, although such cases are likely true for other genic regions (e.g., plastid-derived DNA fragments in mitochondrial genomes; Guo et al., 2017; Hao et al., 2022b). However, the mitogenome assemblies of most Ophioglossaceae genera are still incomplete, and thus mitochondrial MORFFO might be overlooked. Besides, IGT might also occur between organellar and nuclear genomes (see below). Complete mitogenome or even nuclear genome sequences from different Ophioglossaceae genera in future may shed light on the origins of these MORFFO genes.

Regardless of mitochondrial or plastid origins, MORFFO sequences from Ophioglossaceae are usually grouped together (**Figures 4**, **5**, **S2, S3**). Similar phylogenetic patterns can be found in MORRFOs of other fern families, too (**Figures S2, S3**). In addition, relationships inside these family clusters are sometimes consistent with their species tree (e.g., FTOL; Nitta et al., 2022). However, such closely related MORFFO sequences are usually found in different genic locations. For instance, in the Ophioglossaceae cluster with only plastomic *morffo2*, these sequences are in various plastomic locations, including intergenic regions of *rps4-trnL*, *trnF-ndhJ*, and *trnT-trnfM* in LSC, and *ndhF-rpl21* in SSC (cluster III in **Figure 5**). In addition, some plastomes host two diverged copies, such as *morffo2* in *Helminthostachys* (**Figure 5**). These patterns imply that MORFFOs can be inherited vertically, and replicate to switch their positions. One plausible scenario to explain their varied genic distributions among close relatives but with a moderate sequence similarity is that these organellar MORFFOs are derived from IGT of different nuclear duplicates. We highlight this possibility for some fern cases because several *morffo2*-like sequences were also found in the nuclear genome of the tree fern *Alsophila spinulosa* (Hook.) R.M.Tryon (**Figures 5**, **S3**), which was recently whole-genome-sequenced (Huang et al., 2022).

In this study, we also inferred phylogenetic relationships for *morffo1* and *morffo2* by incorporating a broad sequence sampling from the GenBank nucleotide collection (**Figures 4**, **5**, **S2, S3**). Notably, we found MORFFO sequences not only in fern plastomes, but also in their mitogenomes, organellar genomes of other non-angiosperm green plant lineages, and genomes of viruses and phages. In these MORFFO phylogenies, fern sequences are the most dominant ones, and they also form a major clade (**Figures 4**, **5**). Interestingly, despite the fact that sequences from the same family always clustered together, we didn’t find an interfamily relationship highly matching our current understanding of the fern tree of life (**Figures S2, S3**). Moreover, in some highly supported clades, sequences from a distantly related family can be found nested within. For example, two of three Ophioglossaceae sequence clusters of *morffo2* are placed in the basal positions of the fern-dominant lineage (clusters II and III in **Figure 5**), but another one is imbedded in sequences of leptosporangiate ferns, and also contains a sequence from the leptosporangiate family Pteridaceae (i.e., *Myriopteris scabra* (C.Chr.) Grusz & Windham; in cluster I in **Figure 5**). These apparently random interfamily relationships imply the possibility of frequent fern-to-fern HGTs for the MORFFO genes. We also noticed that similar phenomena are revealed in some HGT cases reported previously in ferns (i.e., *PHY3* and *Tma12*) but they are found in nuclear genomes instead (Li et al., 2014; Li et al., 2018).

### 4.3 Origins of mitochondrial HGTs in Ophioglossaceae

Due to the recent increase in the description of fern mitogenome sequences, more HGT cases have been identified from these mitogenomes (e.g., Zumkeller et al., 2023). In Ophioglossaceae, besides two previously identified cases (i.e., *matR* and *nad1* in *Botrypus*), we identified more mitochondrial HGTs in more genera (**Table 2**). We also confirmed their presence in multiple conspecific samples (data not shown), so excluding the possibility of individual sample contamination. These HGTs are very likely located in mitogenomes because their read coverages match those of other mitochondrial genes (**Table 2**, **S6**). Moreover, some of them are linked with mitochondrial genes from Ophioglossaceae hosts (**Table 2**). Phylogenetically, these HGTs in Ophioglossaceae mitogenomes are derived from either angiosperm or fern donors. In some species, we can find HGTs with multiple origins (**Table 2**). Interestingly, among the cases with angiosperm origins, Santalales species were found to be the major donor, and root-parasitic species were most likely ones as HGT sequences are all grouped with root-parasitic lineages such as *Comandra* and *Santalum*, *Arjona* and *Schoefpia*, and *Malania*. In *Sahashia*, two HGT genes are likely derived from a different root-parasitic lineage in the angiosperm order Lamiales—Orobanchaceae (**Figure 6A**). These findings imply that several Ophioglossaceae taxa live intimately with root-parasitic angiosperms during some stages of their life history. Despite these angiosperm parasites not having been reported to directly host on a fern, these angiosperms are possible to contact with ferns indirectly via mycorrhizal fungi (Brundrett, 2002; Heide-Jørgensen, 2010; de Vega et al., 2011). Such a scenario is plausible especially for Ophioglossaceae ferns because they are always symbiotic with mycorrhizal fungi in their life history that starts from a mycoheterotrophic gametophyte generation to a sporophyte generation relying on mycorrhizae (Lang, 1902; Winther and Friedman, 2007; Whittier, 2015; Chen et al., 2022). In addition, two mitochondrial HGTs in *Mankyua* were found to originate from other Ophioglossaceae ferns, most likely *Sceptridium* in subfamily Botrychioideae; and the others were from non-Ophioglossaceae ferns (**Table 2**; **Figure S4**). Fern-to-fern HGTs were also proposed in previous studies, and gametophytes of these plants are suggested to be the most vulnerable stage of being genetically “transformed” (Wickell and Li, 2020). However, it is still difficult to explicitly determine the taxon identity of these donors because fern mitochondrial sequences remain scarce in GenBank and thus only limited fern taxa are analyzed in our mitochondrial phylogenies. Identifying the donors of these HGTs in the future will be very insightful to study mechanisms behind these fern-to-fern and angiosperm-to-fern HGTs.

The unexpectedly common HGTs in Ophioglossaceae raise another question: Do these foreign genes provide any evolutionary advantages or disadvantages to ferns? *PHY3* and *Tma12* are two typical HGT cases in ferns that are usually interpreted to significantly benefit the adaptation of these plants (Li et al., 2014; Li et al., 2018). However, most mitochondrial HGTs presented here appear to be neutral for their Ophioglossaceae hosts, because they are pseudogene-like and/or redundant copies in the host mitogenomes (**Table 2**). Nonetheless, two HGTs, the *ccmB* in *Mankyua* and the *rps12* in *Sahashia*, may potentially to complement the functional losses of native copies in the host mitogenomes (**Table 2**). Another intriguing case is the *atp9* copies in the *Helminthostachys* mitogenome. In addition to the native copy, *Helminthostachys* acquired two HGT copies respectively from a non-Ophioglossaceae fern and an angiosperm, and both foreign copies appear to be functional. Further assessment of their expression profile would be helpful to answer whether these HGTs function like beneficial genes.

### 4.4 Evolutionary trends of organellar genomics in subfamily Ophioglossoideae

One of the notable evolutionary trends in Ophioglossoideae is its acceleration in changes in organellar genomes. From our phylogenomic tree, an obvious rate elevation at the DNA level can be found in Ophioglossoideae, which exhibits longer branches than other lineages (**Figure 3**). Moreover, their plastomes have evolved to become AT-rich and reduced in size. The most significant case is the genus *Ophioderma*, and its plastome is the smallest known by far in ferns that is ~0.123 Mbp in size (**Table S1**; Du et al., 2019; Du et al., 2021; Du et al., 2022). We can find similar trends also in the mitogenomes of these Ophioglossoideae genera. Apparently, these taxa have reduced mitogenomes, in which *ccm* genes and several introns are lost (**Figure 3**; **Table S5**), and the total DNA amount is also decreased. Mitogenomes of *Ophioglossum* have been fully assembled that are revealed with a single chromosome of a smaller size of 0.37 Mbp (Guo et al., 2017; Hao et al., 2022b). By contrast, mitogenomes from other ferns, including *Psilotum* from the sister family, are typically with multiple chromosomes and total lengths ranging from 0.62 to 1.44 Mbp (Guo et al., 2017; Feng and Wicke, 2022; Zumkeller et al., 2023). In the other Ophioglossaceae subfamilies, the mitogenome sizes seem to be even larger, and that in *Helminthostachys* and *Sceptridium* are estimated at greater than 3.19 and 1.16 Mbp based on our preliminary assemblies (Kuo et al., unpublished data). Additionally, mitogenomes of these other subfamilies are occasionally found to be inserted with pseudogenized HGTs (**Table 2**). Taken together, these findings imply a selective pressure on the organellar genomes in subfamily Ophioglossoideae to reduce in size, a phenomenon not observed in other subfamilies.

We speculate that this evolutionary trend in Ophioglossoideae is associated with (1) its mixotrophic lifestyle and/or (2) extraordinarily large nuclear genomes. The members in this subfamily produce proliferous and budding roots, that are mycorrhizal, fleshy, and rich with storage materials (Petry, 1914; Chrysler, 1941; Peterson and Brisson, 1977). Importantly, these roots can “parasitically” acquire carbohydrates from nearby plants via mycorrhizal fungi (Suetsugu et al., 2020). Thus, when photosynthetic aboveground parts are seasonally dormant, these non-green but active underground organs remain capable of growing and even asexually reproducing. In other words, Ophioglossoideae species likely behave as autotrophic and heterotrophic lifestyles separately in different seasons or developmental stages (i.e., root buds vs. latterly foliage stages) during their sporophyte generation. During the heterotrophic stages of the life cycle, organellar genomes that are smaller and AT-rich are favored due to their ability to replicate more rapidly. Consequently, these smaller genomes are likely the result of genic deletions, which are beneficial for the plants during this phase. As the plants transition to the autotrophic stages, where they engage in photosynthesis and assimilation, organellar genomes with deficit characteristics due to deletion-introduction are subsequently selected out. Such selection from the heterotrophic stages can be much severe if the constructional needs of a nuclear genome become greater (e.g., need more nucleotides for its genome replication), and thus it competes for resources with organellar genomes. This hypothesis can better explain why organellar genome downsizing is evident only in Ophioglossoideae which is diagnostic by huge nuclear genomes. Especially for *Ophioderma*, which has the largest nuclear genome size in this family (**Table 1**), and also exhibits the most diverged organellar genomes (**Tables S1,** **S5**; **Figure 3**).

### 4.5 Conclusion and future perspectives

Using the phylogenomic analyses that apply both plastomic and mitogenomic datasets, sequences from all genera, and fine-tuned substitution models, we provide strong evidence for well-resolved relationships of the subfamilies in Ophioglossaceae. Importantly, this solid subfamily backbone is consistent with many key morphological and genomic changes in the family and supports the systematic positions of Helminthostachyoideae and Mankyuoideae. At the generic level, some relationships within subfamily Ophioglossoideae remain unclear, and its seven genera are sorted into four highly supported clades. To resolve these difficult nodes within Ophioglossoideae, we expect to utilize a larger phylogenomic dataset in the future that includes more generic representatives as well as more loci from the nuclear genomes, such as transcriptomic sequences. In addition, this study also highlights several evolutionary genomics issues in Ophioglossoideae, including its elevated substitution rates in organellar genomes and a trend toward AT-rich and smaller plastomes. A tentative hypothesis is also provided here to explain these interesting patterns in Ophioglossoideae by considering its mixotrophic lifestyle and huge nuclear genomes. In addition, evolutionary rate heterogeneity and AT-biased codon usages in this fern lineage are likely associated with gene expression levels (Hao et al., 2022a). Therefore, future investigation of rate statics and transcriptomic profiles is also critical to study comparative genomics in Ophioglossoideae.

One of our interesting findings here is identifying numerous HGT (or IGT-like) cases in Ophioglossaceae organellar genomes. These HGTs predominantly occur in the mitogenomes of subfamilies Mankyuoideae, Helminthostachyoideae, and Botrychioideae. However, these foreign mitochondrial genes seem to be pseudogenized or functionally redundant for host plants. Notably, the origins of these mitochondrial HGTs can be traced to different vascular plant lineages, and ferns and root-parasitic angiosperms appear to be the most important donors. These findings also imply that, behind such steady genic “transformations”, these Ophioglossaceae ferns establish intermate and frequent connections with root-parasitic angiosperms and ferns during certain stages in their life history. Studying the underlaid mechanisms of these HGTs is promising in order to explore a novel manner of genic transformation in plants, particularly for their organellar genomes which remains difficult for most plants.

Finally, the current study points out the importance of fern mitogenome sequences in studying various issues of evolutionary genomics in plants, including their phylogenomics and HGT/IGT mechanisms. However, mitogenomes remain understudied across fern diversity. To date, complete or near complete mitogenomic sequences have been described in less than six fern genera (Guo et al., 2017; Song et al., 2021; Feng and Wicke, 2022; Hao et al., 2022b; Zumkeller et al., 2023), and this number is even less than those published for nuclear genomes. This lack of fern mitogenomic data is in part due to the complexity of these genomes in most species, which is rich in long repetitive sequences and even IGT from plastomes. As adopted in the current study, assembling strategies with short reads alone can recover most mitochondrial gene sequences for phylogenomic analyses. However, these approaches cannot produce a complete mitogenome assembly in most ferns. The most promising way to sequence a fern mitogenome is to adapt the long-read NGS approaches. For this, both an efficient protocol of high-molecular-weight DNA extraction (e.g., Xie et al., 2023) and a long-read sequencing strategy (e.g., Nanopore) are indispensable. Therefore, we are looking forward to future attempts of long-read sequencing and assembly of fern mitogenomes, which will facilitate publication of mitogenome sequences across fern diversity.

## Data availability statement

The datasets presented in this study can be found in online repositories. The names of the repository/repositories and accession number(s) can be found below: Bioproject accession numbers: PRJNA1019085.

## Author contributions

L-YK: Conceptualization, Data curation, Formal Analysis, Funding acquisition, Investigation, Methodology, Project administration, Resources, Software, Supervision, Validation, Visualization, Writing – original draft, Writing – review & editing. H-JS: Conceptualization, Data curation, Formal Analysis, Investigation, Resources, Validation, Visualization, Writing – review & editing. DK: Funding acquisition, Investigation, Software, Writing – review & editing. P-JX: Data curation, Investigation, Writing – review & editing. CW: Resources, Writing – review & editing. AE: Data curation, Resources, Writing – review & editing. JRG: Conceptualization, Funding acquisition, Resources, Supervision, Writing – review & editing.
