# Supplementary material for: Organellar phylogenomics of Ophioglossaceae fern genera
Source: Front Plant Sci. 2024 Jan 15;14:1294716. doi: 10.3389/fpls.2023.1294716 (PMC10823028; doi:10.3389/fpls.2023.1294716)

FIGURE S1 | Phylogenomic results of the genera under subfamily Ophioglossoideae. Details of the different applied models and datasets can be found in “Materials and Methods” of the main text. ML UFBS = maximum likelihood ultrafast bootstrap.

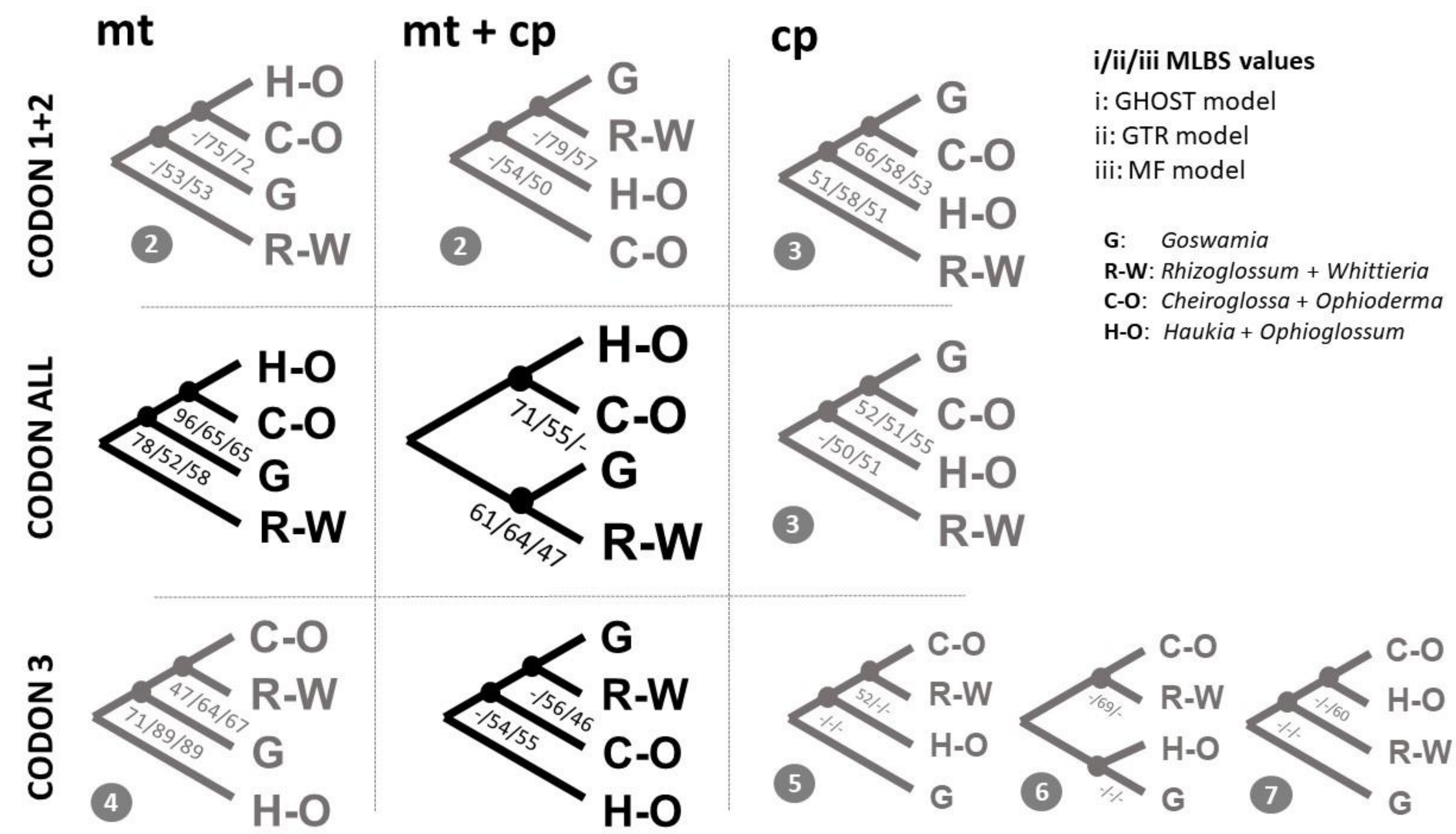

Supplement: Supplementary file 4 [file Image_1.pdf]
