# Supplementary material for: Organellar phylogenomics of Ophioglossaceae fern genera
Source: Front Plant Sci. 2024 Jan 15;14:1294716. doi: 10.3389/fpls.2023.1294716 (PMC10823028; doi:10.3389/fpls.2023.1294716)

FIGURE S3 | Phylogeny of *morffo2*. Each of the tip names includes the following information: genomic origin, GeneBank accession number, and site positions. For the plastomic ones, the genic position is indicated in bold behind their tip names. The Ophioglossaceae samples are indicated by green. The values on the branches are ML UFBS.

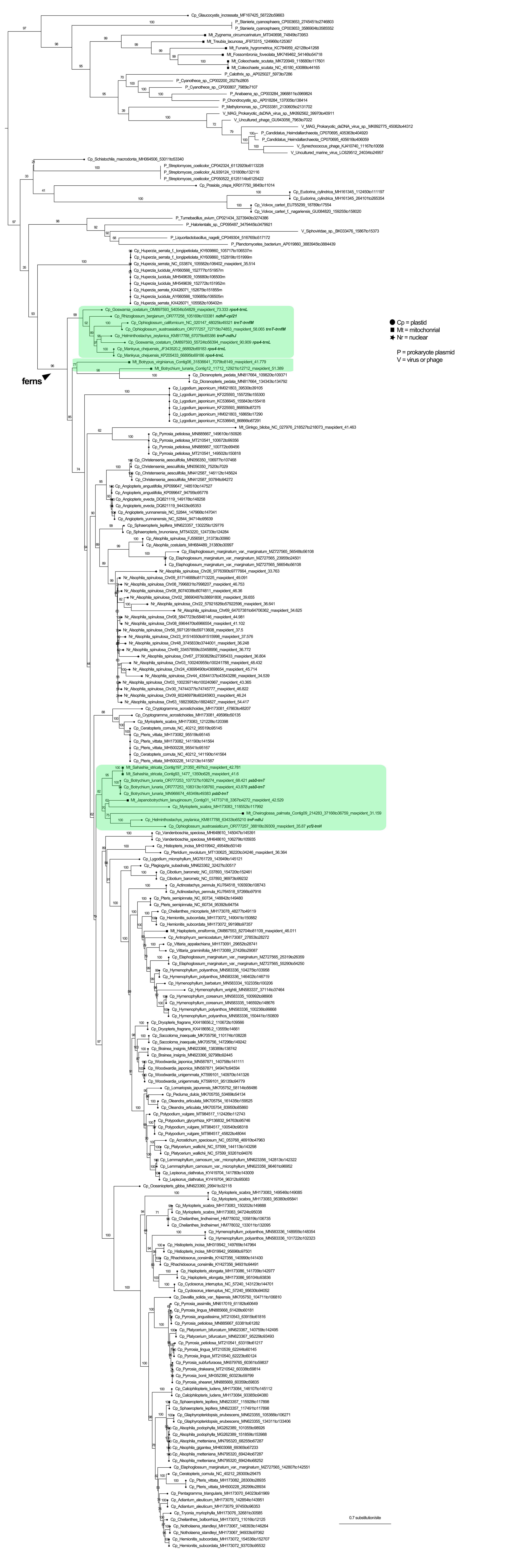

Supplement: Supplementary file 6 [file Image_3.pdf]
