# Supplementary material for: Organellar phylogenomics of Ophioglossaceae fern genera
Source: Front Plant Sci. 2024 Jan 15;14:1294716. doi: 10.3389/fpls.2023.1294716 (PMC10823028; doi:10.3389/fpls.2023.1294716)
Supplement: Supplementary file 7 [file Image_4.pdf]

FIGURE S4-23 | Locus phylogenies for the HGT genes found in Ophioglossaceae mitogenomes. The values below the branches are their ML UFBS.

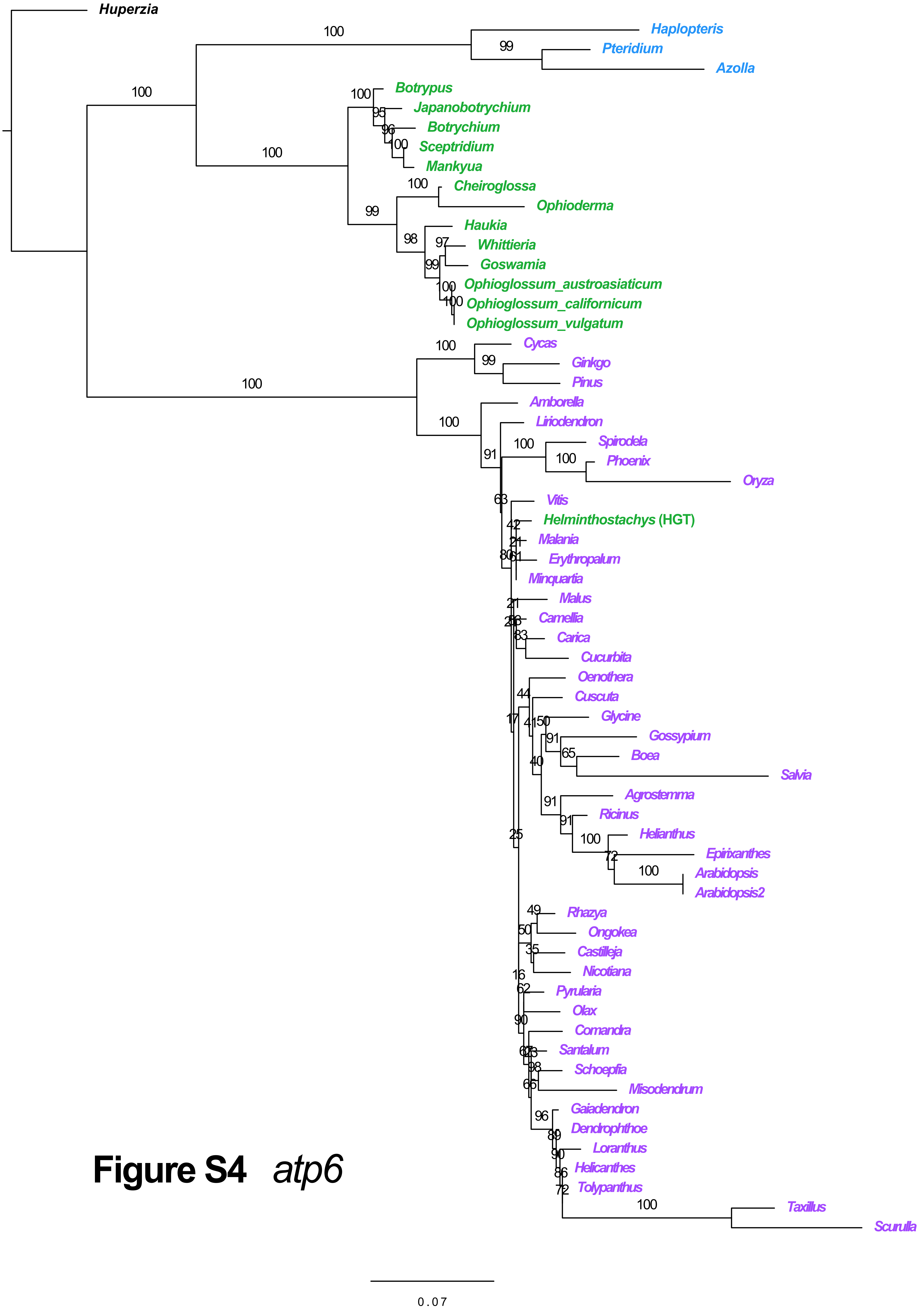

Figure S4 *atp6*

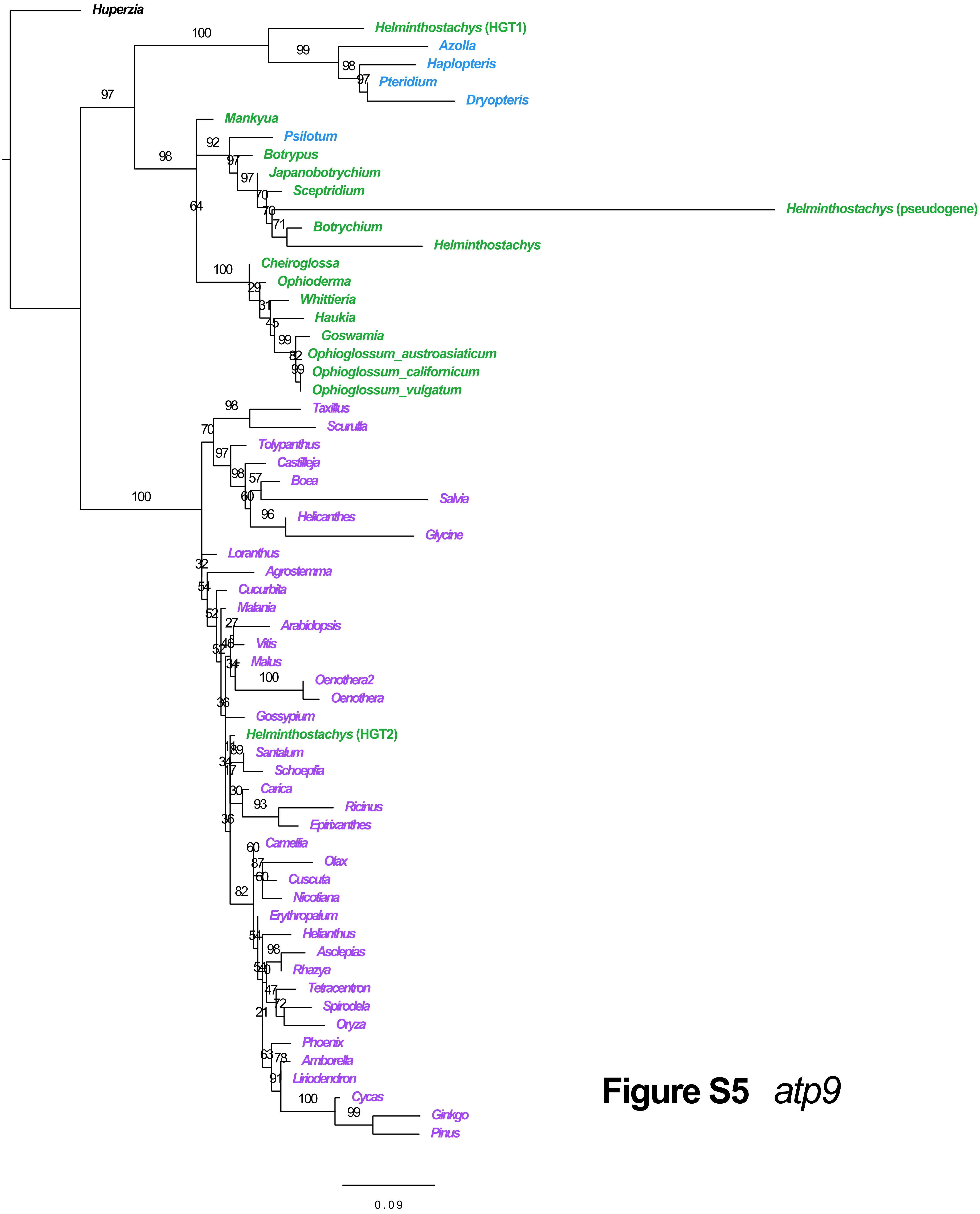

Figure S5 *atp9*

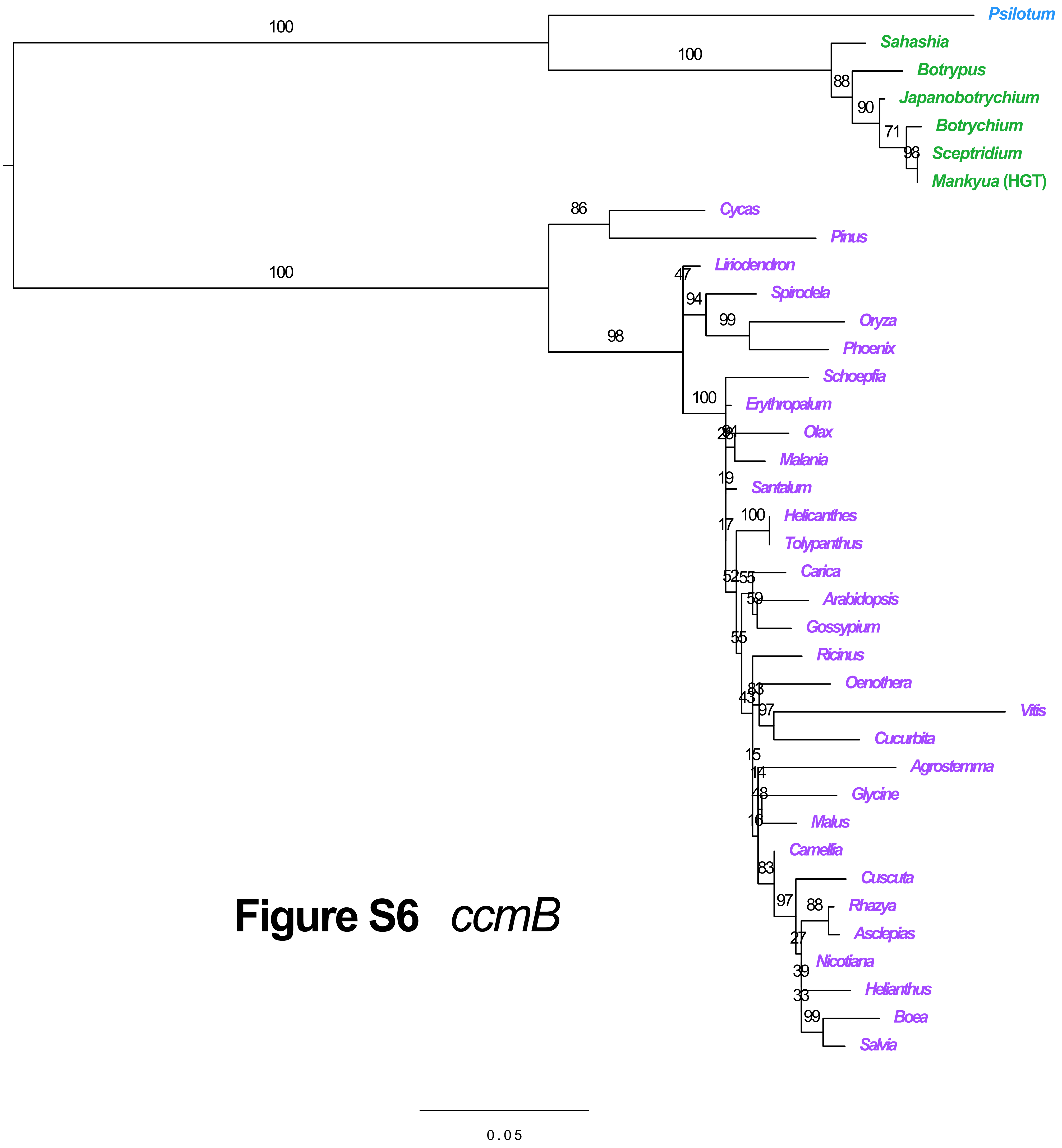

Figure S6 *ccmB*

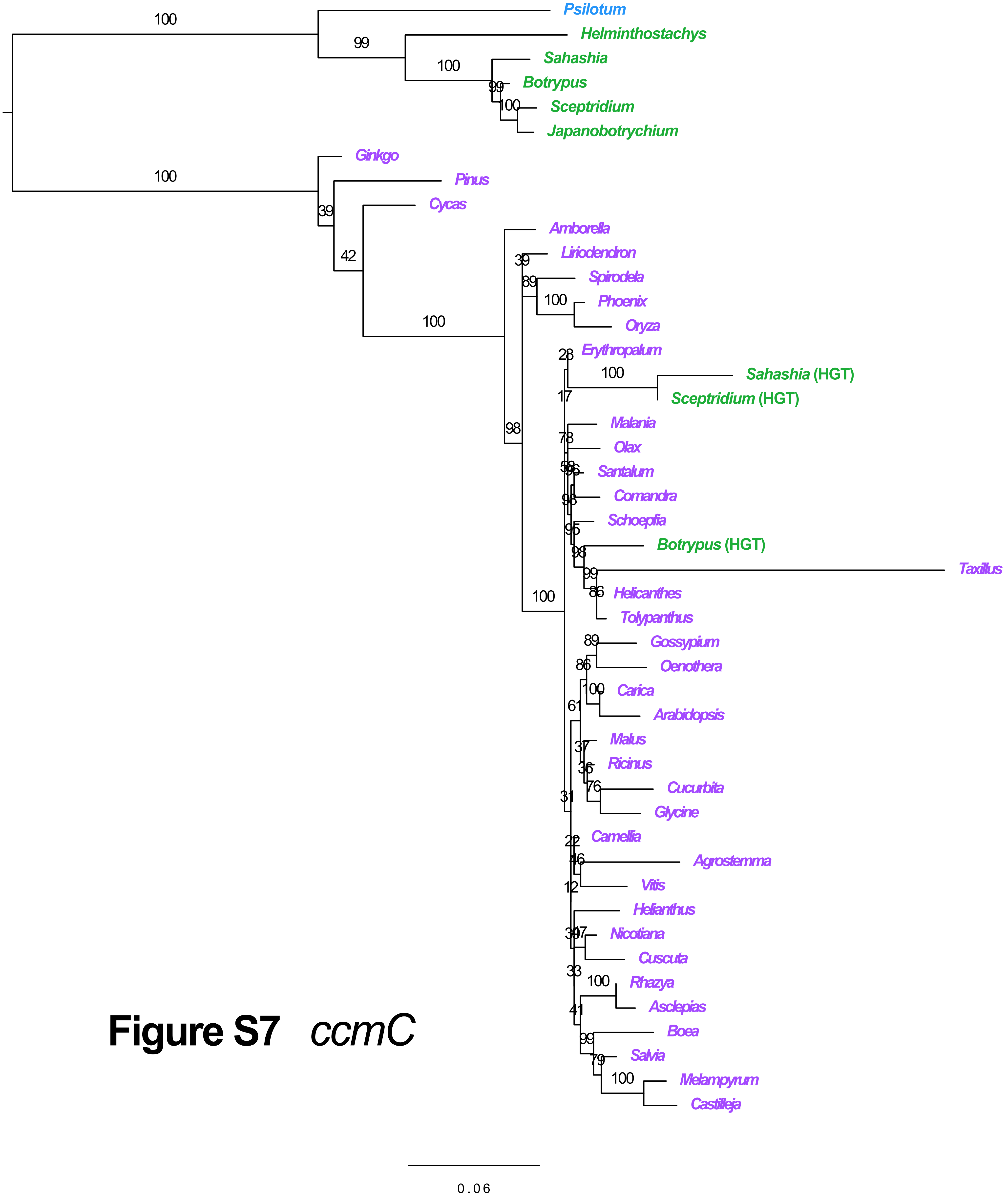

Figure S7 *ccmC*

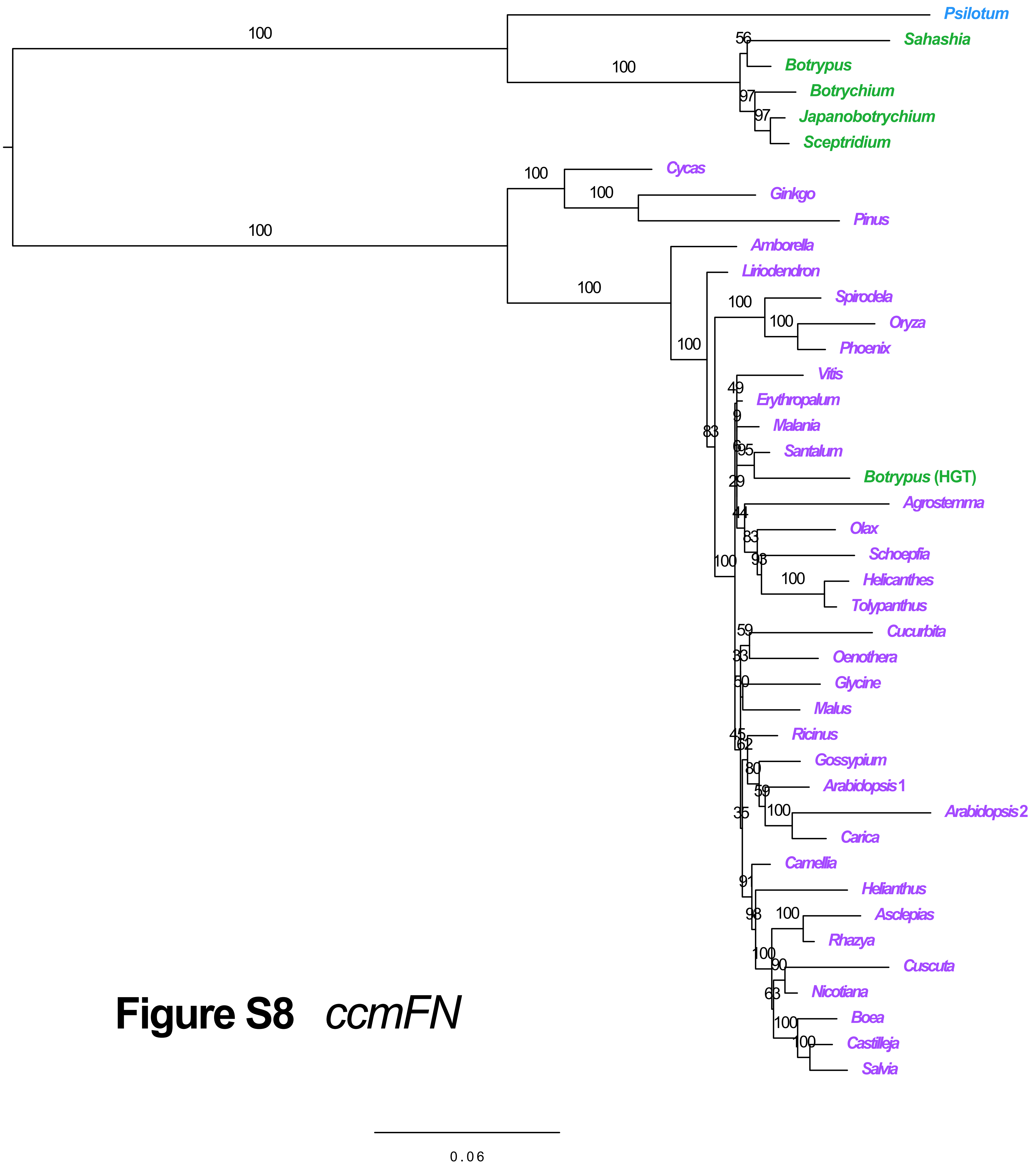

Figure S8 *ccmFN*

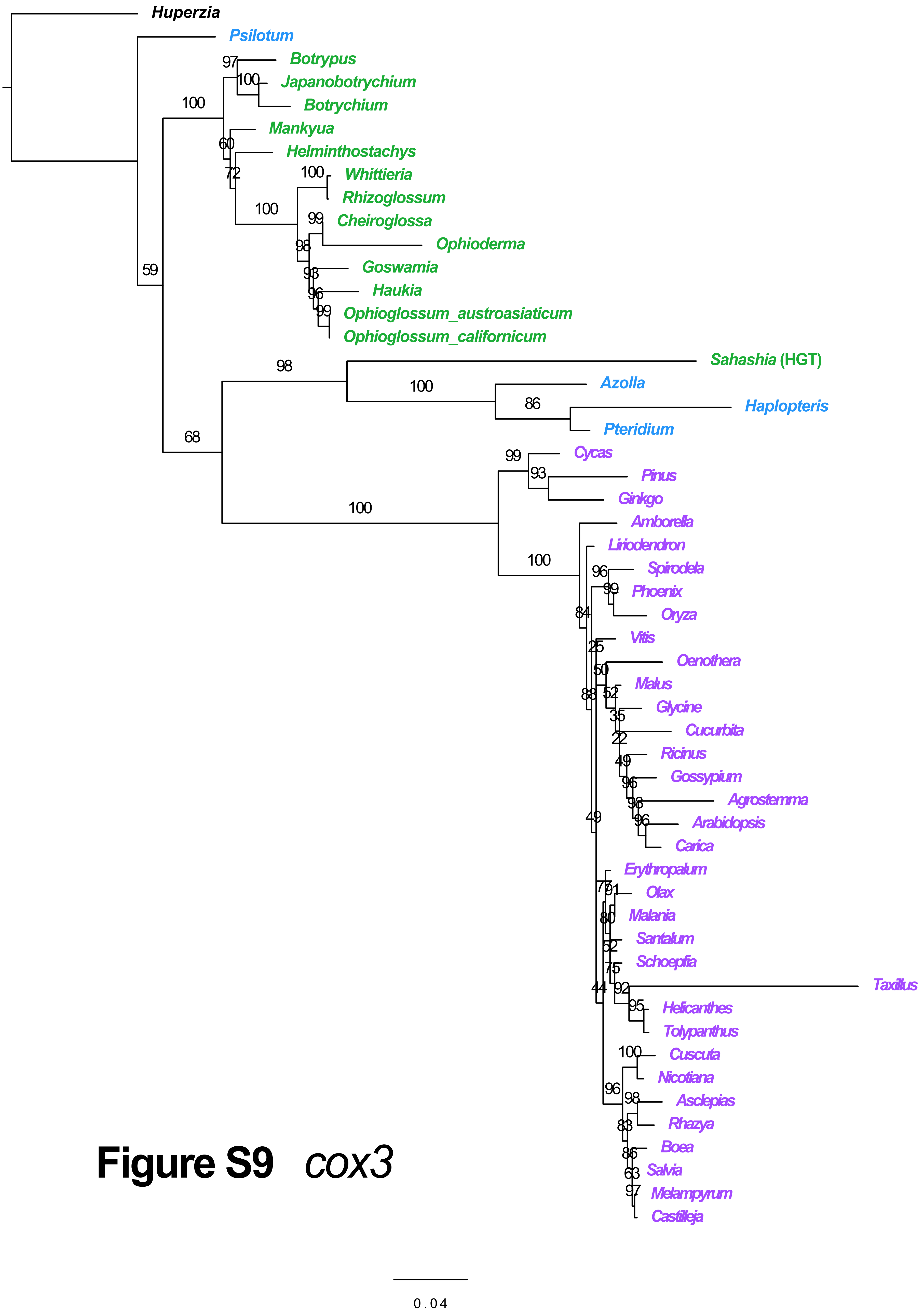

Figure S9 *cox3*

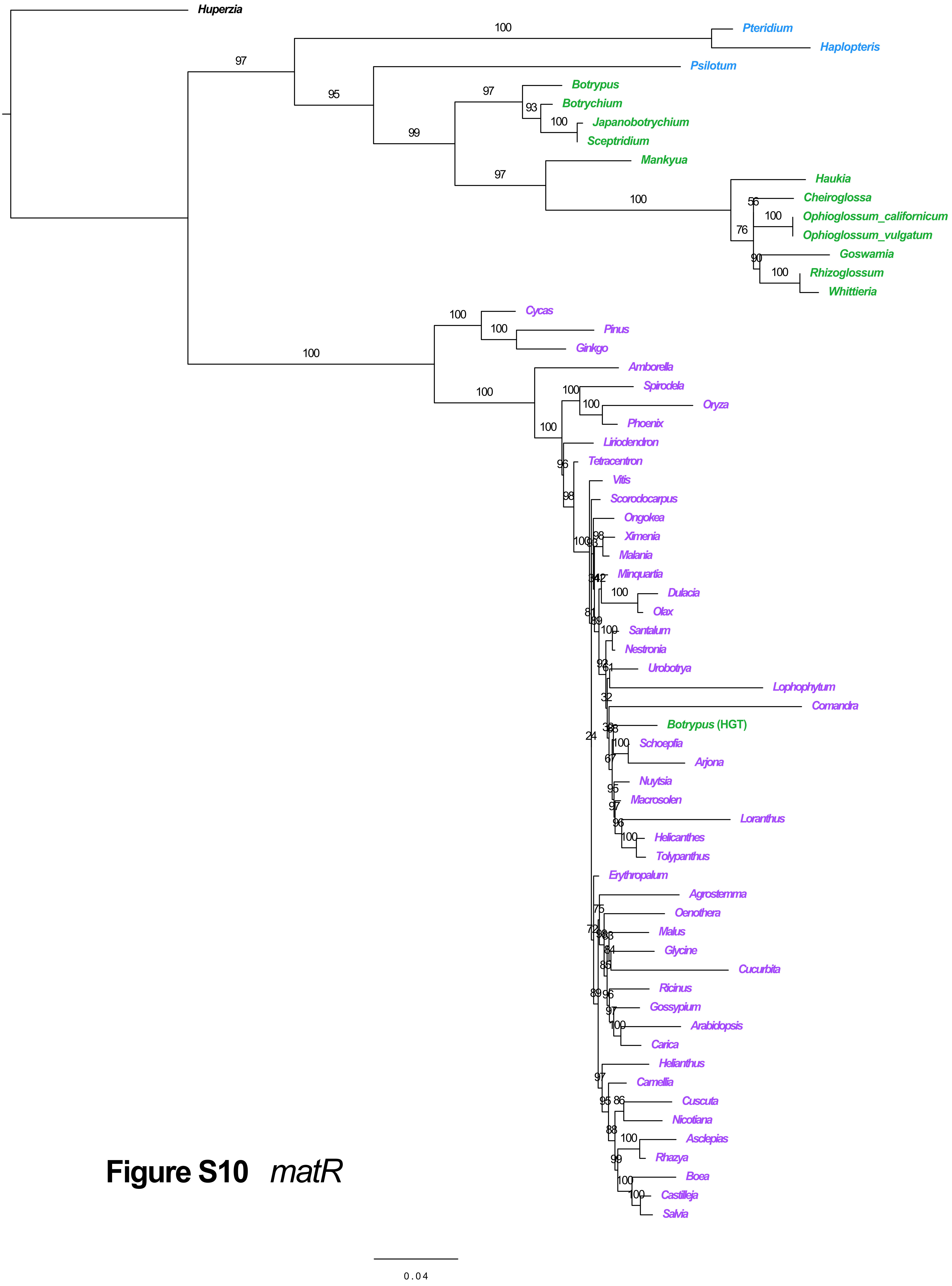

Figure S10 *matR*

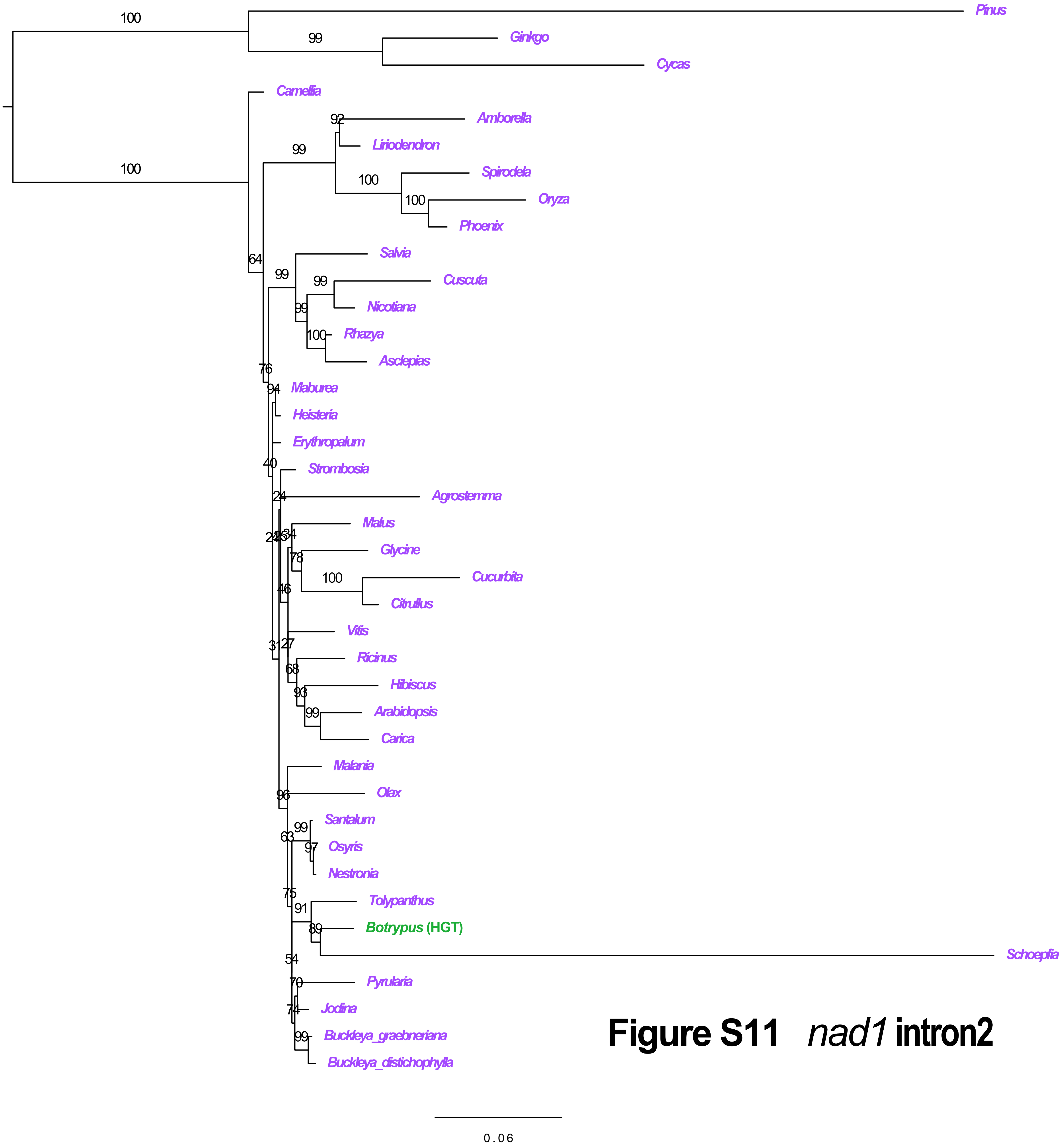

Figure S11 *nad1* **intron2**

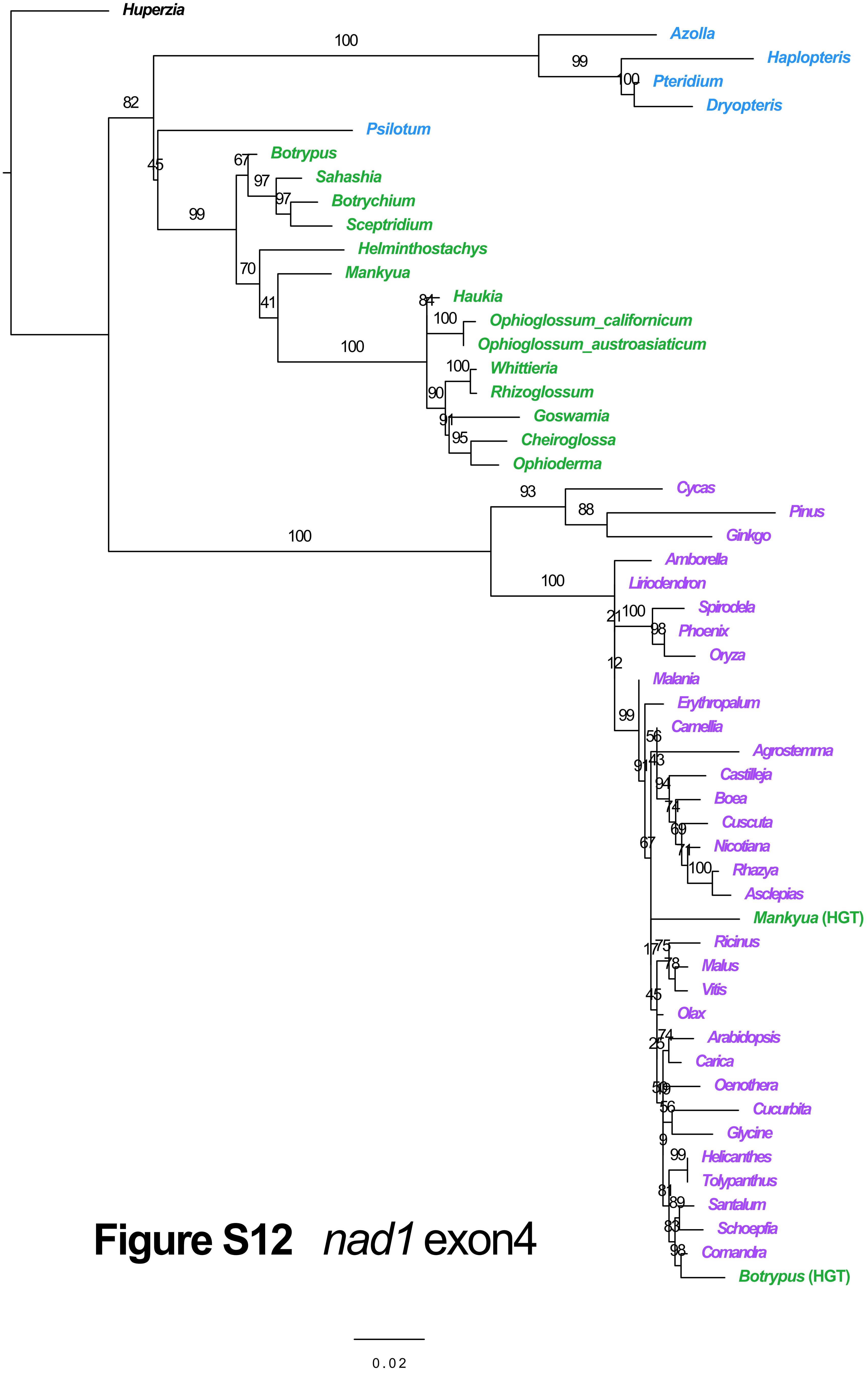

Figure S12 *nad1* exon4

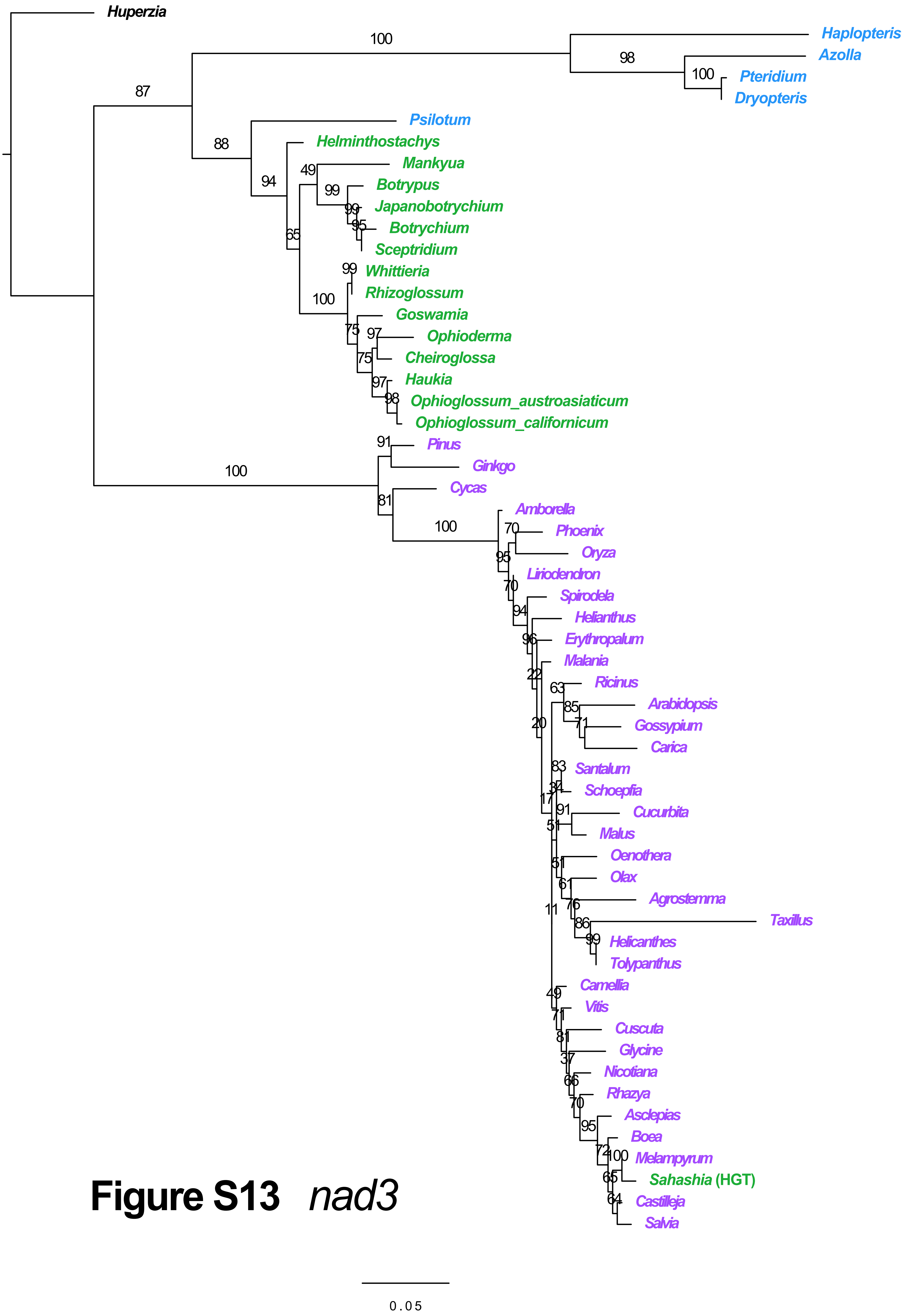

Figure S13 *nad3*

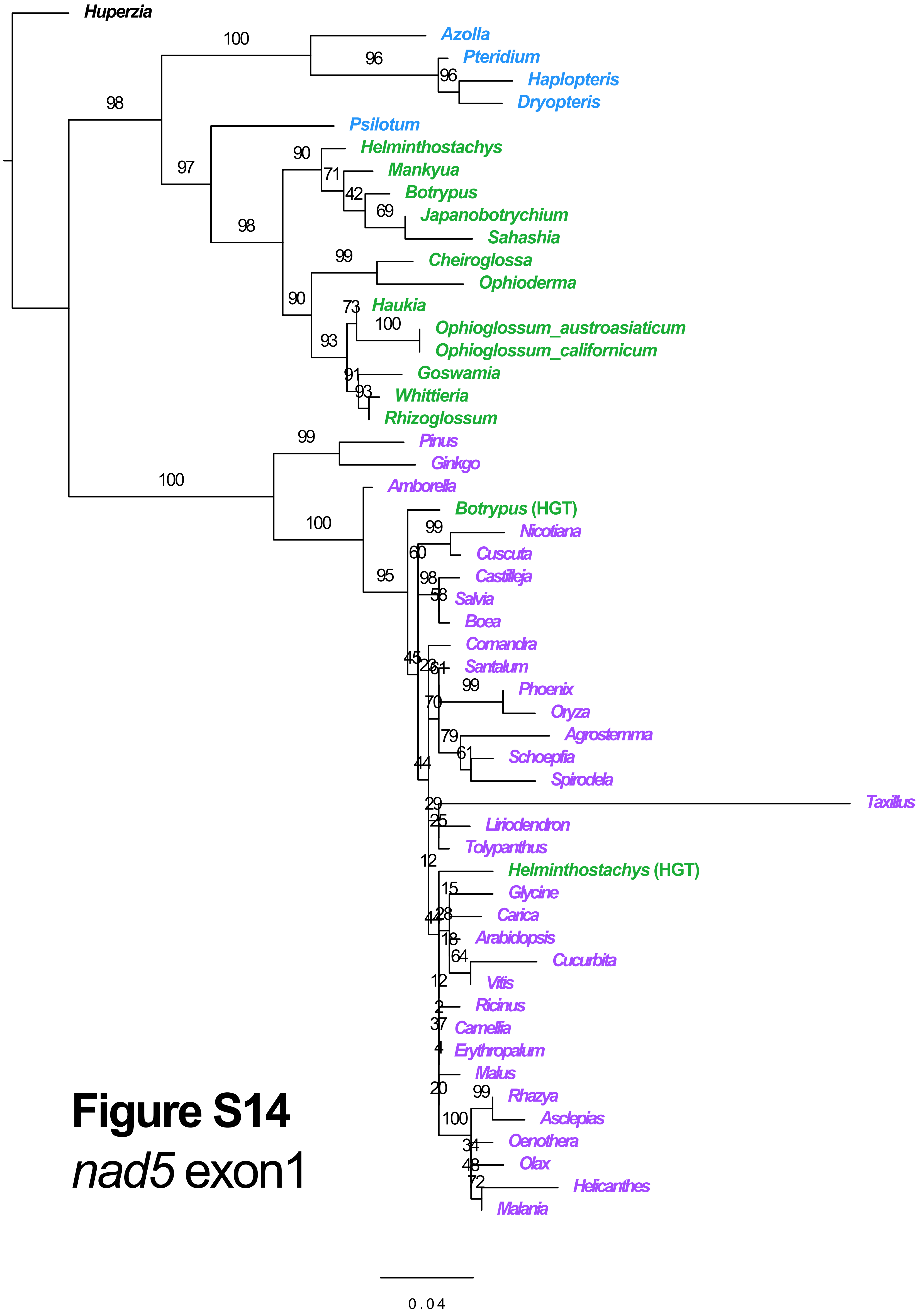

Figure S14
nad5 exon1

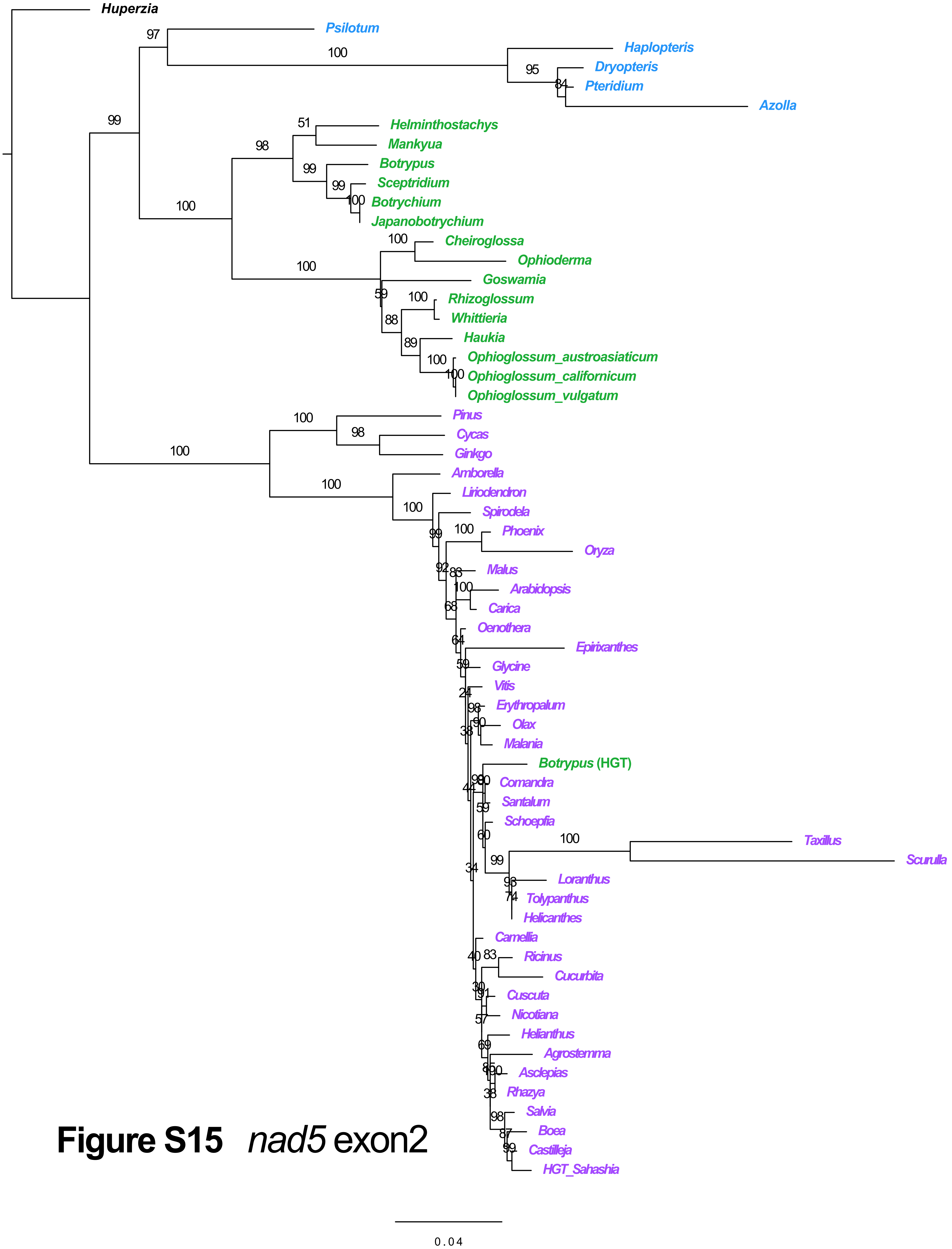

Figure S15 *nad5* exon2

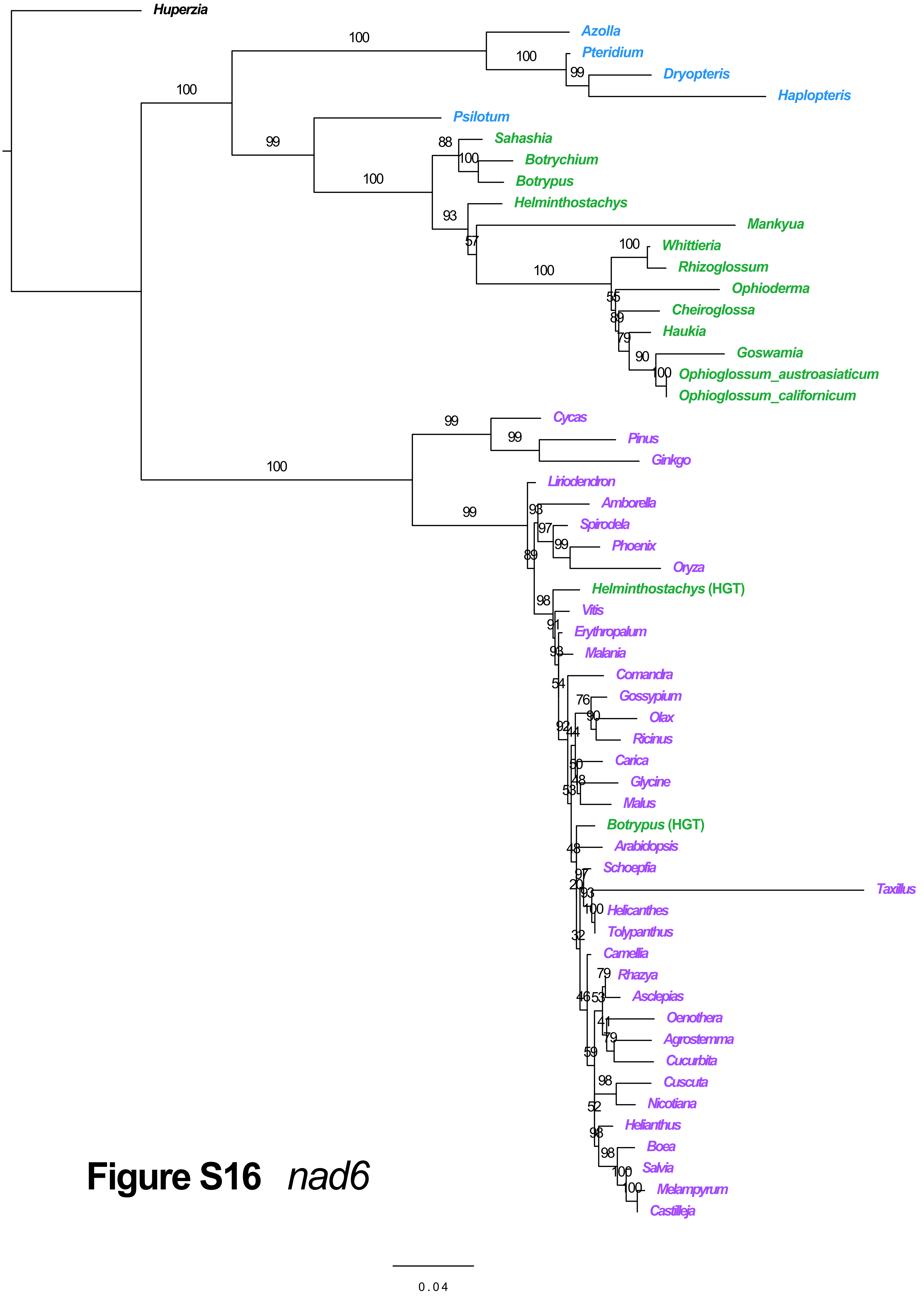

Figure S16 *nad6*

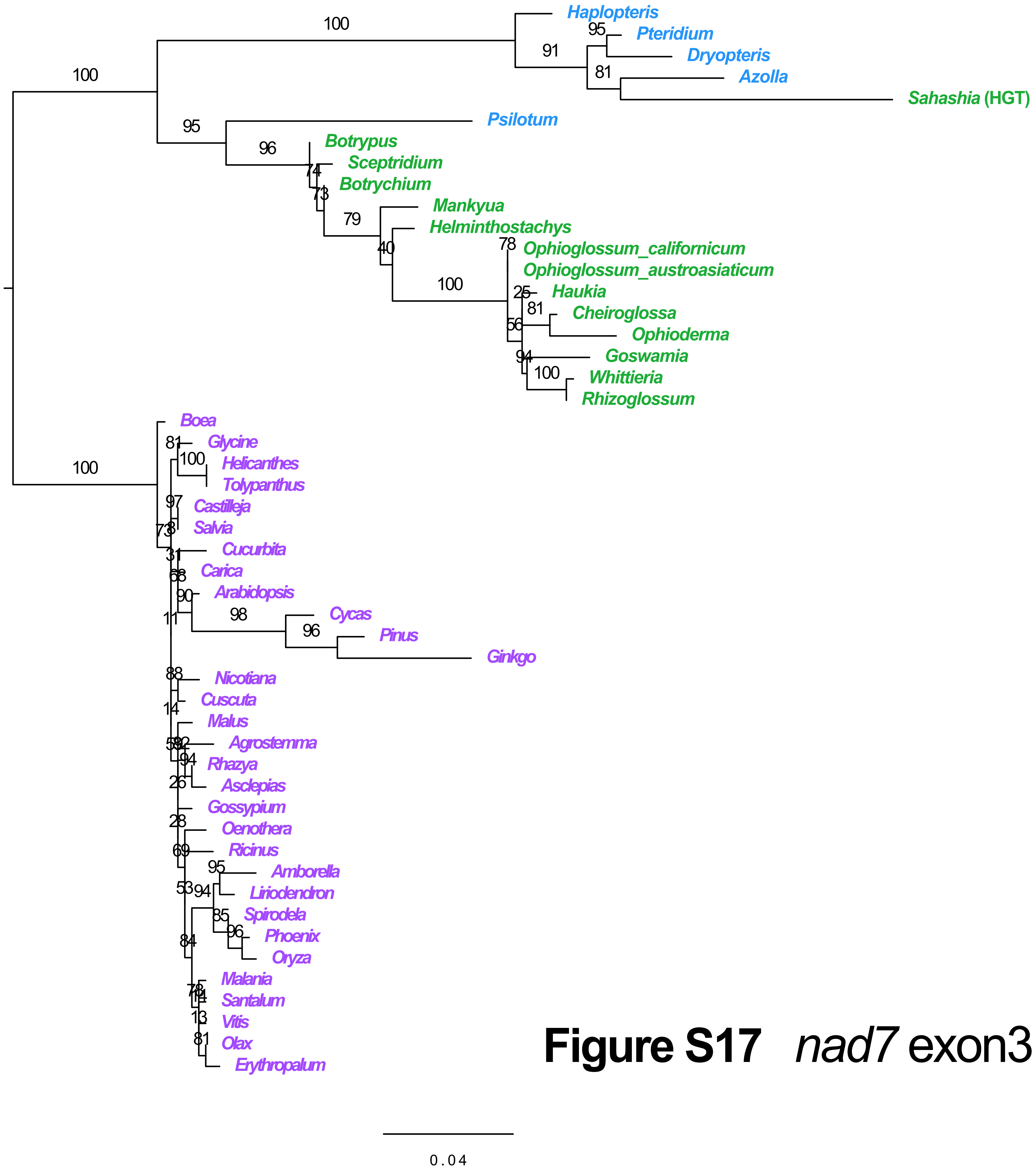

Figure S17 *nad7* exon3

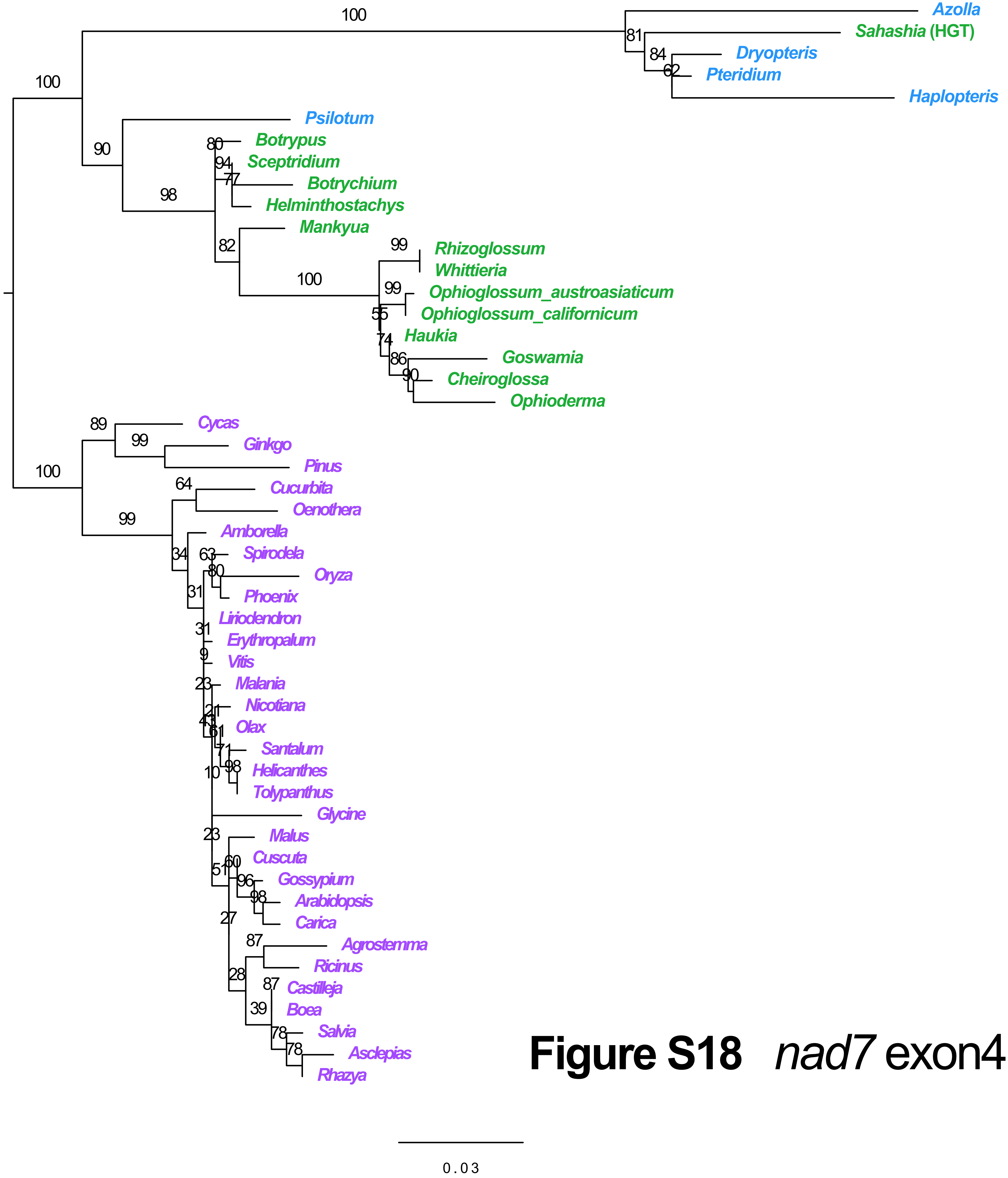

Figure S18 *nad7* exon4 + 5

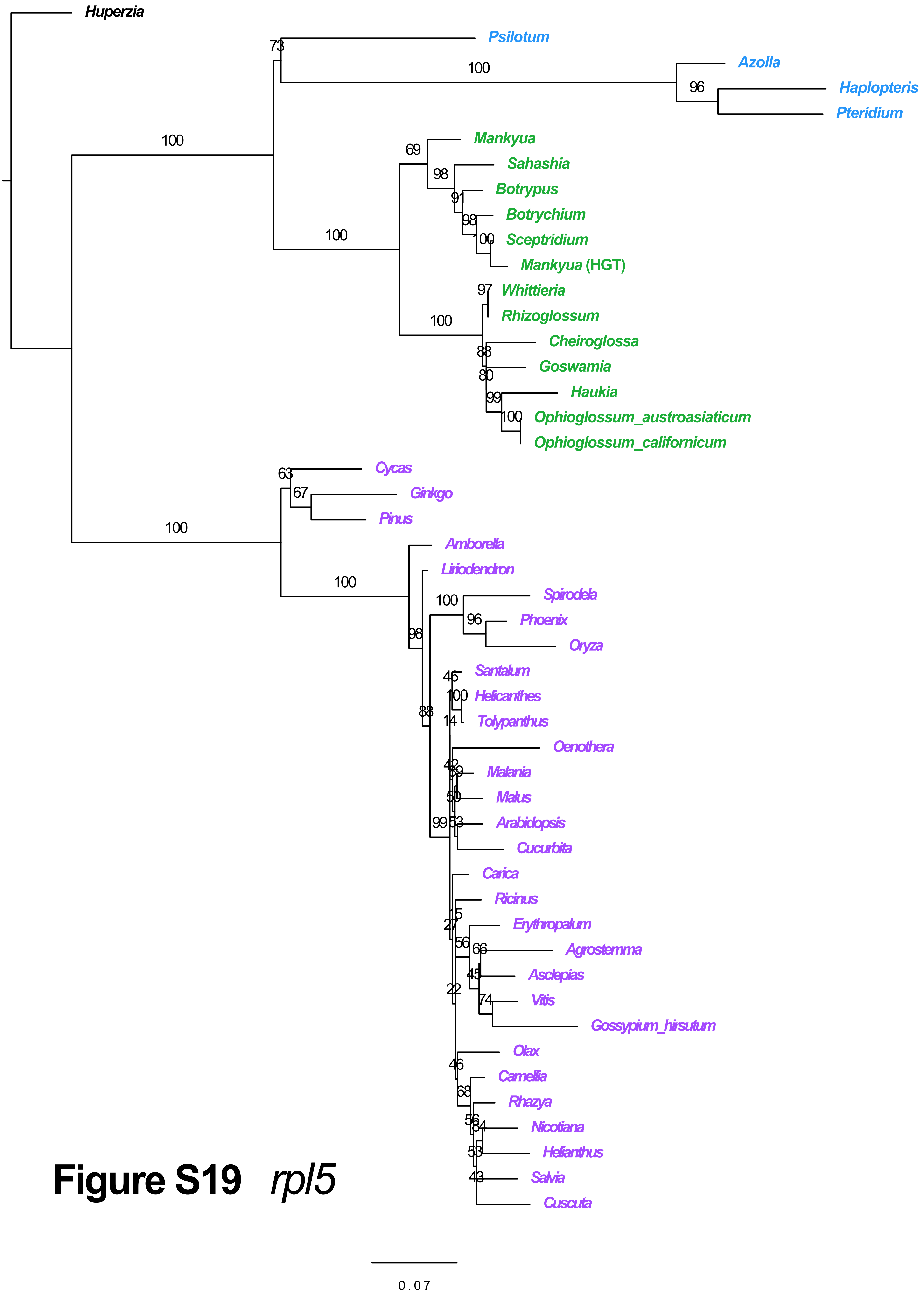

Figure S19 *rpl5*

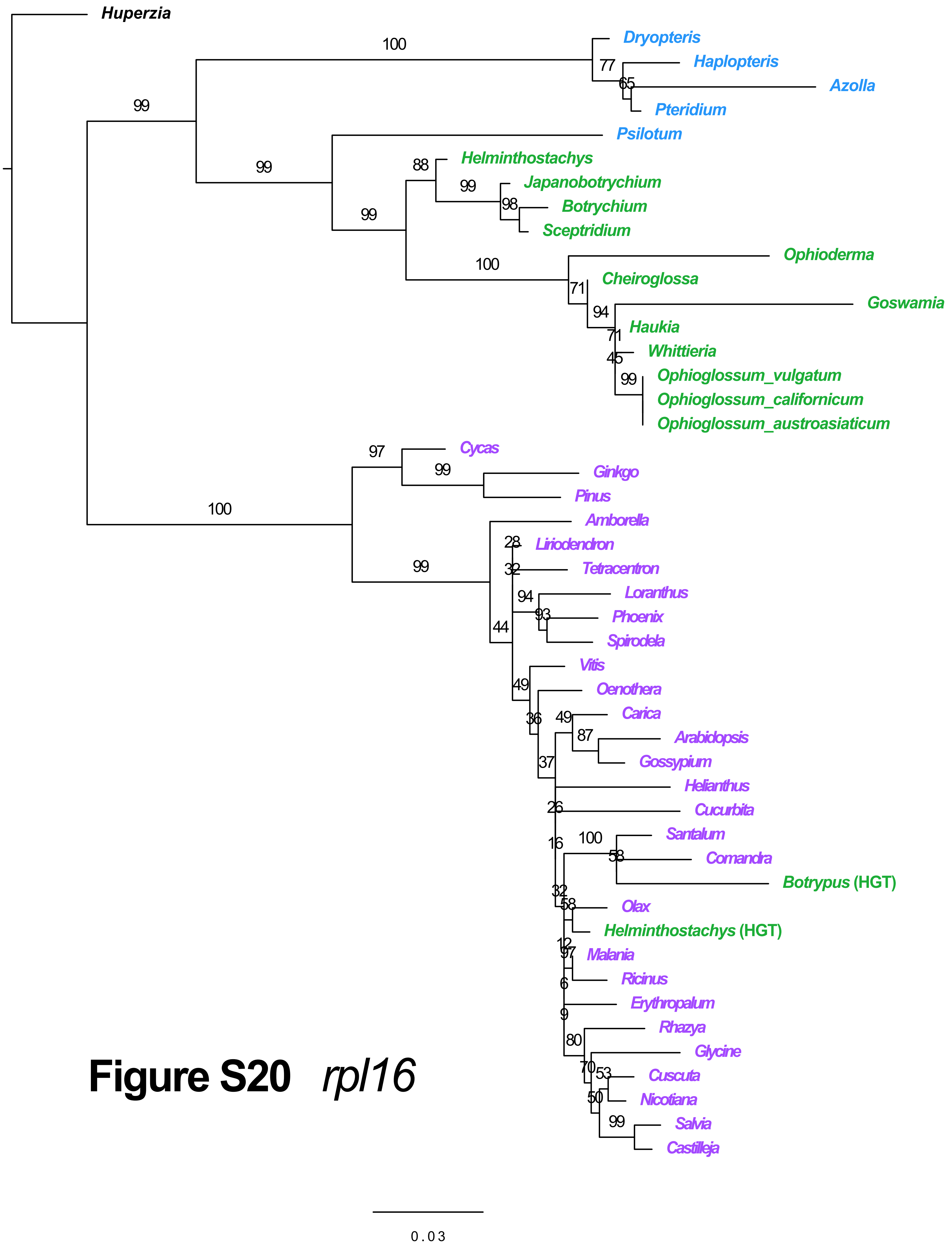

Figure S20 *rpl16*

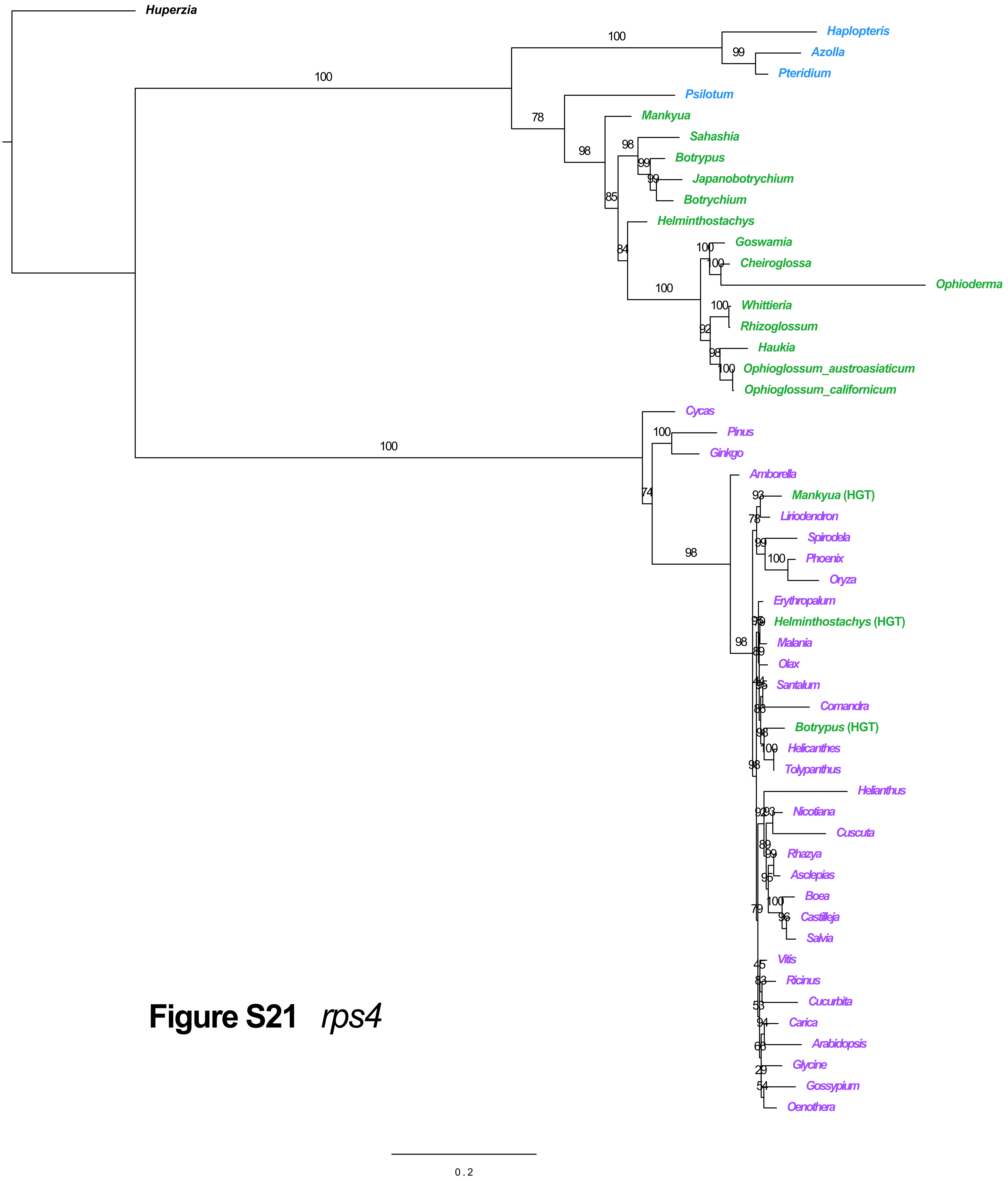

Figure S21 *rps4*

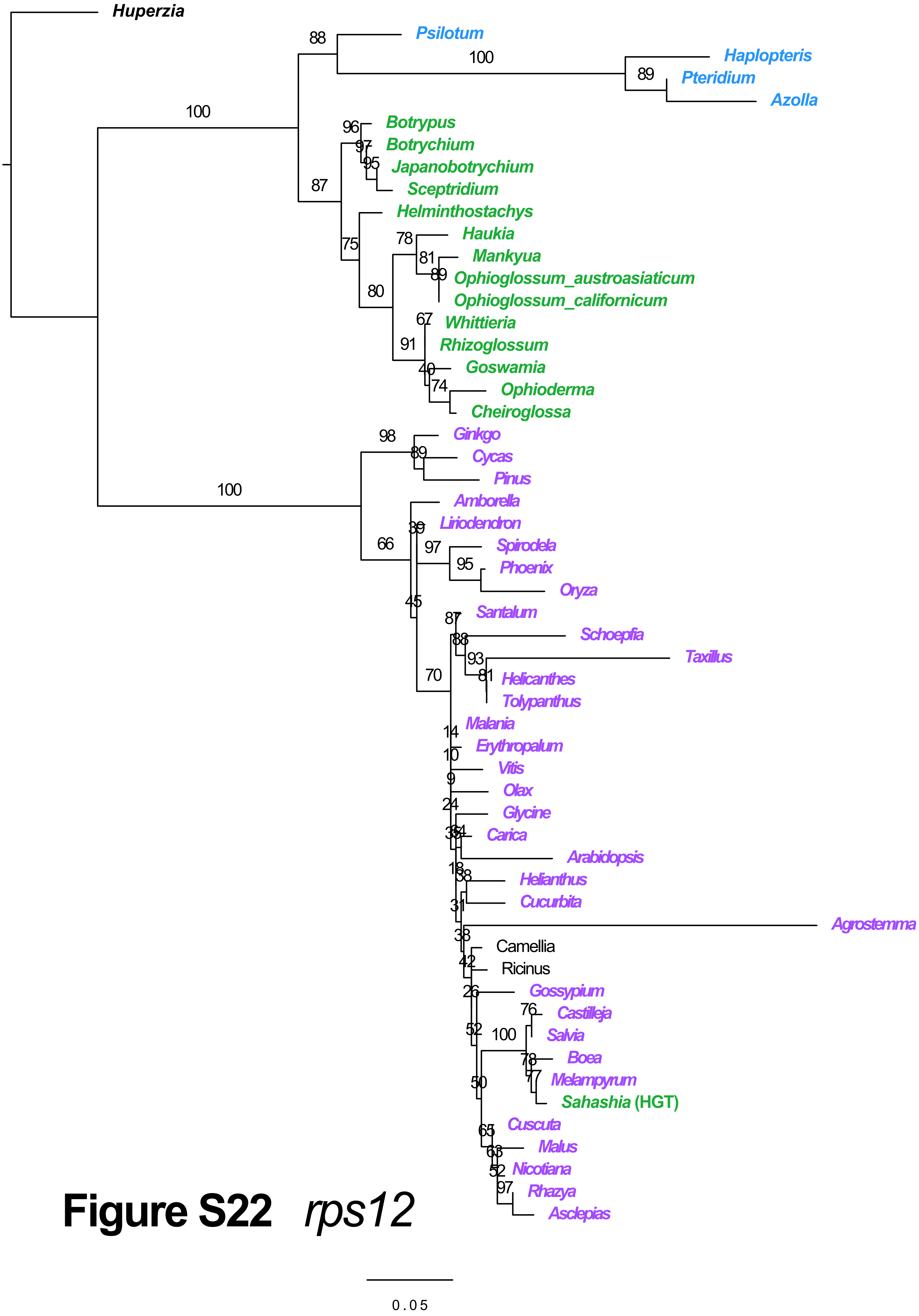

Figure S22 *rps12*

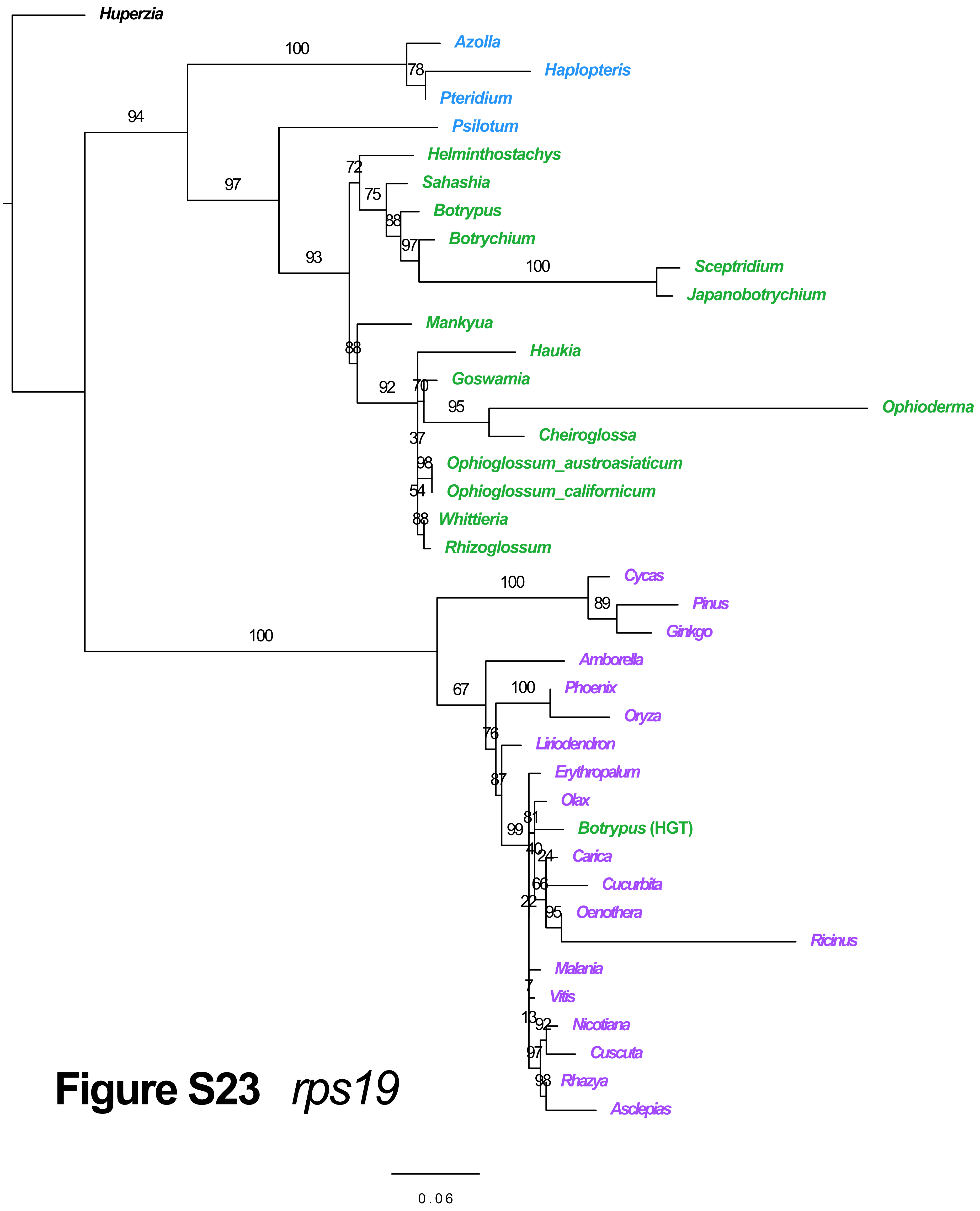

Figure S23 *rps19*
